# Homologous recombination-mediated targeted integration in monkey embryos using TALE nucleases

**DOI:** 10.1186/s12896-018-0494-2

**Published:** 2019-01-15

**Authors:** Chu Chu, Zhaohui Yang, Jiayin Yang, Li Yan, Chenyang Si, Yu Kang, Zhenzhen Chen, Yongchang Chen, Weizhi Ji, Yuyu Niu

**Affiliations:** 1 Yunnan Key Laboratory of Primate Biomedicine Research; Institute of Primate Translational Medicine, Kunming University of Science and Technology, Kunming, 650500 China; 2 The Cardiology Division, Department of Medicine, Li Ka Shing Faculty of Medicine, University of Hong Kong, Hong Kong, SAR China

**Keywords:** Non-human primate, Homologous recombination, TALENs, Gene editing

## Abstract

**Background:**

Non-human primate (NHP) models can closely mimic human physiological functions and are therefore highly valuable in biomedical research. Genome editing is now developing rapidly due to the precision and efficiency offered by engineered site-specific endonuclease-based systems, such as transcription activator-like effector nucleases (TALENs) and the clustered regularly interspaced short palindromic repeats (CRISPR)/CRISPR-associated protein-9 nuclease (Cas9) system. It has been demonstrated that these programmable nucleases can introduce genetic changes in embryos from many species including NHPs. In 2014, we reported the first genetic editing of macaques using TALENs and CRISPR/Cas9. Subsequently, we characterized the phenotype of a methyl CpG binding protein 2 (MECP2)-mutant cynomolgus monkey model of Rett syndrome generated using the TALEN approach. These efforts not only accelerated the advance of modeling genetic diseases in NHPs, but also encouraged us to develop specific gene knock-in monkeys. In this study, we assess the possibility of homologous recombination (HR)-mediated gene replacement using TALENs in monkeys, and generate preimplantation embryos carrying an EmGFP fluorescent reporter constructed in the *OCT4* gene.

**Result:**

We assembled a pair of TALENs specific to the first exon of the *OCT4* gene and constructed a donor vector consisting of the homology arms cloned from the monkey genome DNA, flanking an *EmGFP* cassette. Next, we co-injected the TALENs-coding plasmid and donor plasmid into the cytoplasm of 122 zygotes 6–8 h after fertilization. Sequencing and immunofluorescence revealed that the *OCT4*-*EmGFP* knock-in allele had been successfully generated by TALENs-mediated HR at an efficiency of 11.3% (7 out of 62) or 11.1% (1 out of 9), respectively, in monkey embryos.

**Conclusion:**

We have successfully, for the first time, obtained *OCT4*-*EmGFP* knock-in monkey embryos via HR mediated by TALENs. Our results suggest that gene targeting through TALEN-assisted HR is a useful approach to introduce precise genetic modification in NHPs.

**Electronic supplementary material:**

The online version of this article (10.1186/s12896-018-0494-2) contains supplementary material, which is available to authorized users.

## Background

Although a wide range of organisms, including worms, fruit flies, zebrafish, and rodents, have been used as research tools for most discoveries [1], non-human primates (NHPs) are excellent models to study human disease and develop therapeutic strategies because of their genetic, physiological and neurological similarity to humans [1–5]. Over the years, genetic approaches have been widely applied to model organisms. However, genetic engineering in monkeys was not available until 2001, when retrovirus-based methods were successfully used to generate transgenic monkey models [6]. This seminal work demonstrated that the monkey genome could be genetically modified efficiently. Subsequently, taking advantage of the high efficiency of lentiviral infection, the groups of Li and Sasaki successfully obtained several transgenic monkeys carrying the mutant *HTT* and *GFP* transgenes, respectively [7, 8]. However, while lentiviral transduction has been a powerful and efficient tool in genetic modification of animal genomes, it has often failed to achieve precise editing. The 2011 emergence of new-generation genome-editing methods based on reprogrammable, site-specific endonucleases, especially the clustered regularly interspaced short palindromic repeats (CRISPR)/CRISPR-associated protein-9 nuclease (Cas9) system and transcription activator-like effector nuclease (TALEN), made genome engineering more effective and reliable. Relative construction simplicity as well as precise and efficient genetic modification of target sites associated with these technologies has dramatically advanced genome editing in diverse species [9, 10]. In particular, these methods substantially facilitated the generation of non-human primate (NHP) models of human diseases. Until now, a number of such models have been established, including those of autism spectrum disorder (ASD) [11], Huntington’s disease (HD) [7], Parkinson’s disease (PD) [12], Duchenne muscular dystrophy (DMD) [13], and, most recently, Rett syndrome [3].

TALENs are fusions of the endonuclease domain consisting of the FokI restriction enzyme, which is only active as a dimer, and the engineered DNA-binding domain comprised by TAL effector (TALE) repeats. These highly conserved repeats are derived from naturally-occurring TALEs of the plant pathogen *Xanthomonas*, which contribute to disease or trigger defense by binding the host DNA and activating effector-specific host genes [14] .

**Fig. 1:**
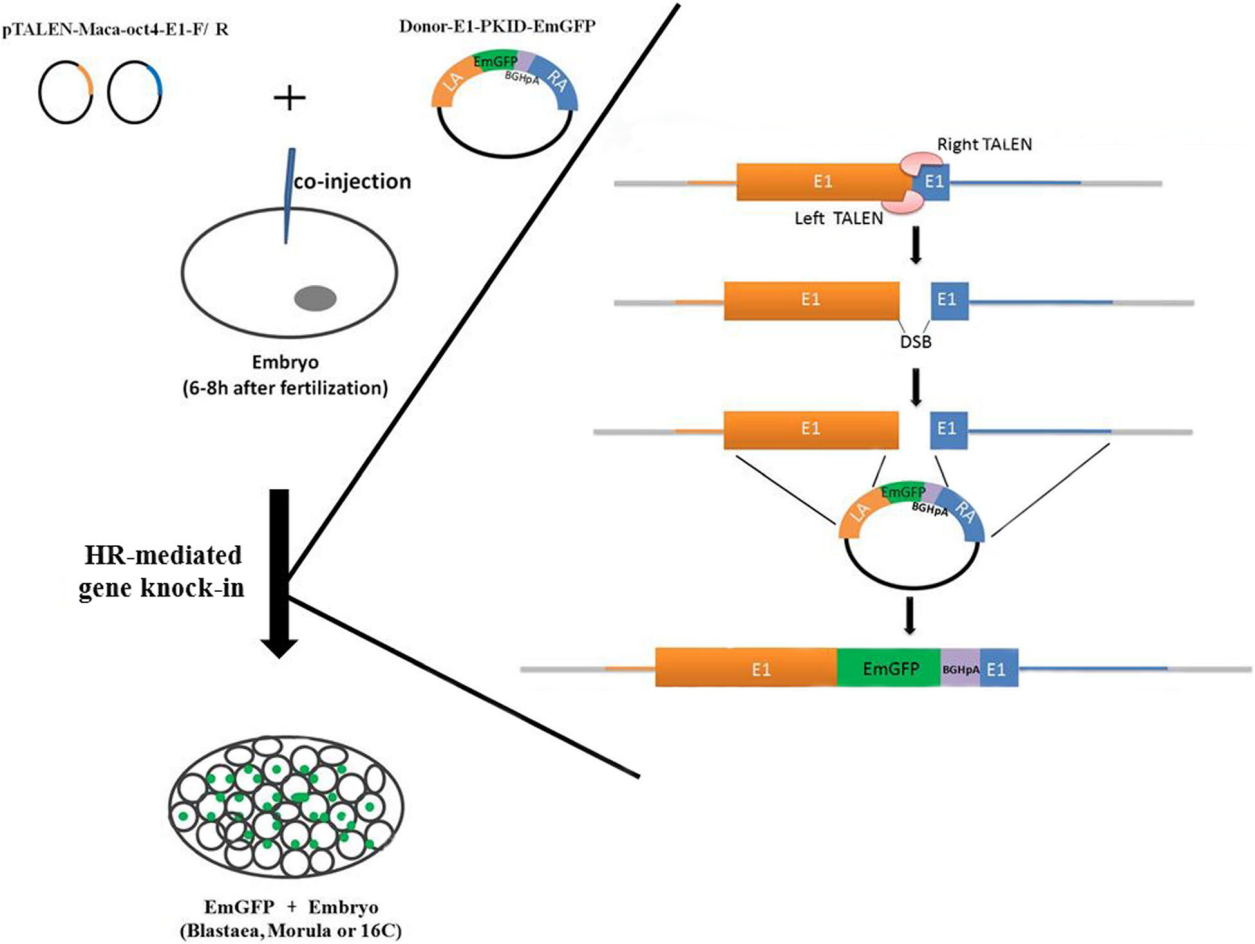
Workflow of TALEN-mediated generation of a monkey embryo carrying an EmGFP reporter in the OCT4 gene. TALENs-coding plasmids, pTALEN-Maca-oct4-E1-F/R, and the donor vector Donor-E1-PKID-EmGFP that targets exon 1 of the *OCT4* gene were designed and co-injected into the cytoplasm of a zygote 6–8 h after fertilization. Treated embryos at the blastocyst, morula and 16-cell stages were collected and analyzed

TALENs can easily modify genomic DNA by introducing double-strand breaks (DSBs) at the desired site, stimulating the cellular DNA repair system. The main components of this system are non-homologous end-joining (NHEJ) and homologous recombination (HR) that result in knock-outs and knock-ins, respectively. NHEJ is a stochastic and error-prone repair process that does not require a repair template and introduces random small insertions or deletions at DNA breakpoints [15]. Gene editing by this system has been used in mammalian cells to disrupt genes, delete chromosomal segments, or restore aberrant reading frames [14]. HR, on the other hand, is a precise repair pathway that requires a DNA template, which harbors a homologous sequence corresponding to the genomic DNA at the DSB site. HR has been used to correct certain mutations associated with diseases such as epidermolysis bullosa [16], sickle cell disease [17, 18], beta-thalassemia [19], and alpha-1 antitrypsin deficiency [20]. The efficiency of gene correction strategies based solely on HR is closely linked to the genomic target, cell type, cell-cycle state, organism species, and efficient delivery of an exogenous repair template. Unlike NHEJ, which occurs during the whole cell cycle, HR is only active during the late S/G2 phases, making HR-dependent gene knock-ins more difficult to achieve compared to the gene knock-outs mediated by NHEJ. Currently, HR-mediated knock-ins have been generated in many species including zebrafish [21], mice [22], rabbit [23], goat [24], sheep [25], cattle [26] and pig [27]. Recently, the groups of Yang and Niu successfully used CRISPR/Cas9-assisted HR to obtain knock-in cynomolgus monkeys with a reporter gene system [28, 29]. Here, we demonstrate TALEN-assisted, HR-mediated generation of monkey preimplantation embryos carrying an EmGFP fluorescent reporter constructed in the *OCT4* gene (Fig. 1). Our results demonstrate that TALEN-based HR is a feasible approach to generate knock-in NHP models.

## Results

### Construction of TALENs and evaluation of their activity in human cells

**Fig. 2:**
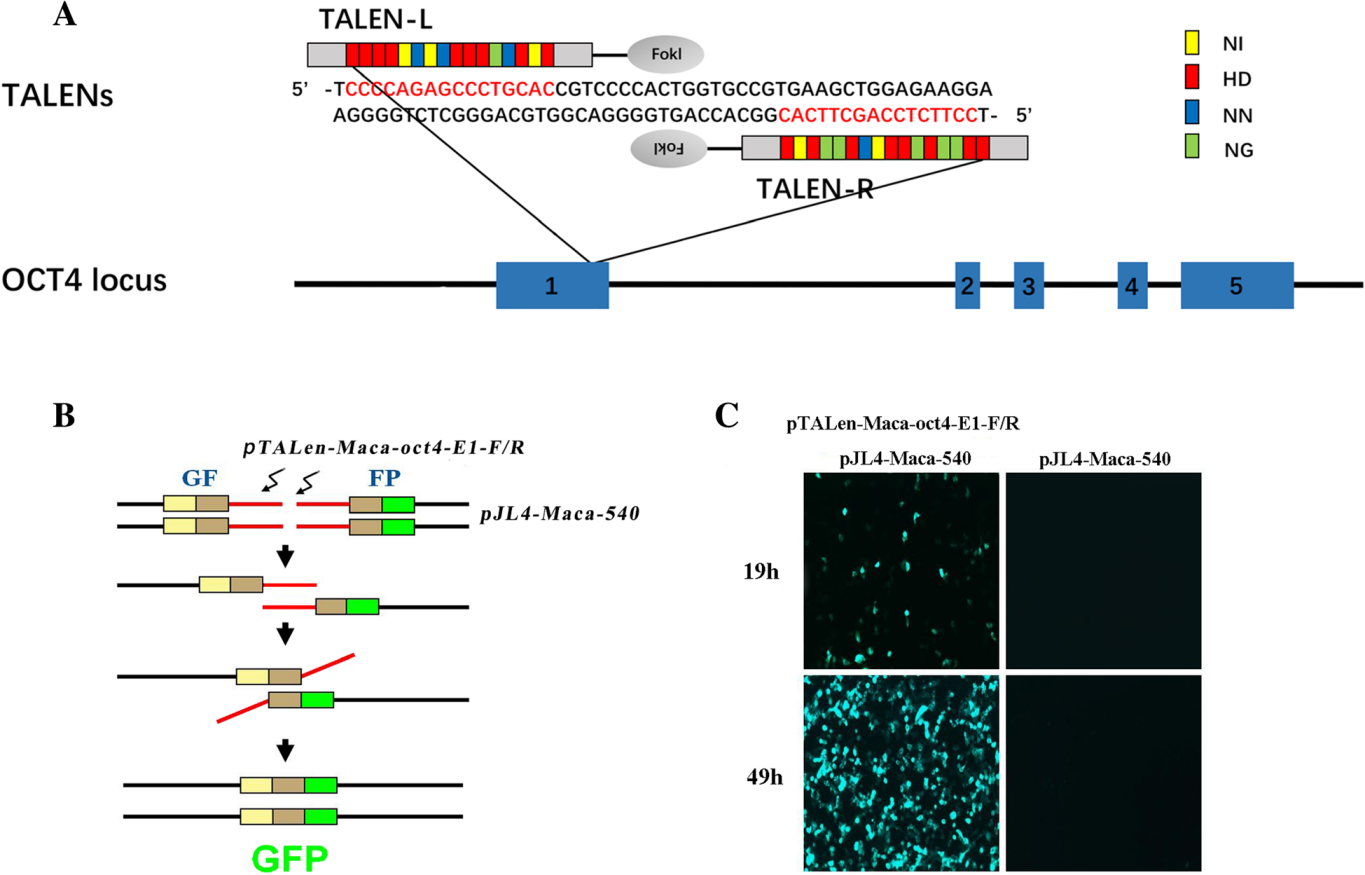
Schematic overview of the OCT4-EI-TALEN construction and the SSA assay for testing the OCT4-TALEN-coding plasmids. **a** The TALEN-targeted sequences within exon 1 of the *OCT4* gene are labeled in red. Assembled Repeat Variable Diresidue (RVD) repeats are represented schematically as boxes and labeled in yellow, red, blue and green. **b** The overview of the GFP-reporter system for SSA. Red lines represent 541 bp oligonucleotides near the TALEN-targeted sites amplified from genomic DNA. **c** Fluorescence intensity of GFP in 293 T cells 19 h or 49 h post transfection with OCT4-E1-TALEN-coding plasmids and the reporter vector pJL4-SSA (left) or controls transfected only with the reporter plasmid (right)

The monkey *OCT4* gene contains 5 exons, similar to that of humans and mice (monkey *OCT4* Gene ID: 714760, human *OCT4* Gene ID: 5460, mice *OCT4* gene ID: 18999, from https://www.ncbi.nlm.nih.gov/gene/?term=OCT4). We designed a pair of TALEN constructs to target exon 1 (Fig. 2a), which is the efficient site for inserting a reporter gene to indicate the expression of OCT4 [30, 31]. These sites are conserved in humans, so we initially used the single strand annealing (SSA) assay in 293 T cells to evaluate the cutting efficiency of Oct4-E1-TALENs based on the GFP expression level, namely the percentage of GFP positive cells (Fig. 2b). Expression of GFP could be observed in 293 T cells transfected with the Oct4-E1-TALEN-coding plasmids, pTALEN-Maca-oct4-E1-F /R, and the reporter vector pJL4-Maca-540 both after 19 h and 49 h, but not in the control group transfected only with the reporter plasmid (Fig. 2c). These results showed that the TALENs were designed and constructed to effectively induce DSBs at the target sites in the human genome, suggesting that this pair of TALENs would work in a monkey as well because of high genomic homology.

### Development of monkey embryos microinjected with the constructed plasmids

The plasmids coding for TALENs, pTALEN-Maca-oct4-E1-F /R, and the donor vector, Donor-E1-PKID-EmGFP, were mixed and injected into the cytoplasm of 122 zygotes 6–8 h after fertilization using a microinjection system under standard conditions. Based on the number of zygotes that were successfully injected, 94.3% (115 of 122), 78.7% (96 of 122), 58.2% (71 of 122), 47.7% (57 of 122), and 40.2% (49 of 122) of the TALENs-treated embryos progressed to the 2-cell, 8-cell, 16-cell, morula, and blastocyst stage, respectively (Table 1). The developmental competence of embryos injected with TALENs was normal, judging by the percentage of embryos that reached the blastocyst stage (40.2%). These results are similar to our previous report for those embryos derived from in vitro fertilization (IVF) [2], indicating that this system is safe and does not influence the development of NHP embryos.

**Table 1:** In vitro developmental potential of the TALENs-injected embryos

Group	Number of embryos	Development stage reached (%)				
		2-cell	8-cell	16-cell	morula	blastocyst
Injected	122	115 (94.3)	96 (78.7)	71 (58.2)	57 (47.7)	49 (40.2)
Control	34	34 (100)	30 (88.2)	23 (67.64)	17 ( 50)	13 (38)

### Genotyping to confirm the targeted gene integration

**Fig. 3:**
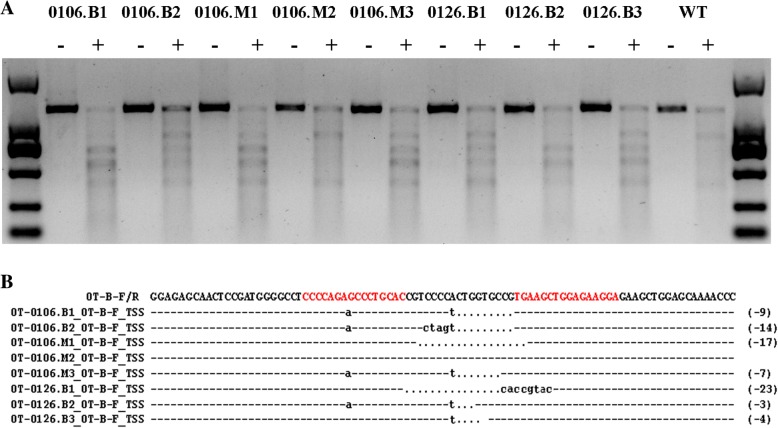
Detection of TALEN-induced mutations in the genomes of monkey embryos. **a** The T7EN1 cleavage assay for on-target efficiency of TALENs .The positive cleavage bands were visible in 7 samples in addition to 0106.M2. **b** Sequencing results showed different indels at the target site. The two short base sequences in red are the target sites for the TALEN pair, while the 17 bp between them comprise the region cleaved by FokI

To test the efficiency of TALENs, the genomic DNA was isolated from the embryos with normal development at the 16-cell, morula and blastocyst stages, respectively, and screened for the presence of site-specific gene modification by PCR amplification of the regions surrounding the target sites, as well as by the T7EN1 cleavage assay (Fig. 3a). The PCR products corresponding to the target sites were amplified using the OT-F/R primers (Table 2) from individual embryos (*n* = 8) in the morula or blastocyst stage and then sequenced. The genomes of up to 87% (7/8) of monkey embryos injected with TALEN-coding plasmids were modified at the target site, visualized by the cleavage bands in the target gene (Fig. 3a). Further characterization of the cleavage by sequencing indicated different indels at the target site with variable mutation size (Fig. 3b). This data demonstrated that the TALENs could also function effectively in monkey embryos.

**Table 2:** Primers used for genotyping

Primer	Sequence (5′ to 3′)	Usage	Amplicon(bp)
L1-F	AGGATCGCTTGAGGACAGGAATT	PCR	3259 bp (5’gDNA~ 3’gDNA)
L1-R	GGAGCCATCACAGGAGACAGAAAA	PCR/sequencing	
S1-F	GATGCATTGAGGGATAGCTCCACACACACATTC	PCR/sequencing	1795 bp (5’ gDNA ~EmGFP)
S1-R	AGGACCATGTGATCGCGCTTCT	PCR	
S2-F	CAGAAGAACGGCATCAAG	PCR	2109 bp (EmGFP~ 3’gDNA)
S2-R	AGTAGGGAGGAAGCAGTAGAA	PCR	
G-F	CACAAGTTCAGCGTGTCCG	PCR	421 bp (EmGFP)
G-R	AGTTCACCTTGATGCCGTTC	PCR	
LI-W1R-G08	ATCCCTCAGCAGCTCTACATTT	sequencing	
L1-W2R-H11	CAGCTTCACGGCACCAGTAC	sequencing	
L1-W3R-C04	GTAGCGGGCGAAGCACTG	sequencing	
S12-W1R-C01	TCCGAGGATCAACCCAGC	sequencing	
OT-F	CACCACCATTAGGCAAAC	PCR	1201 bp target site included
OT-R	GAGGCTGAAGTCAATCAAAA	PCR	

**Fig. 4:**
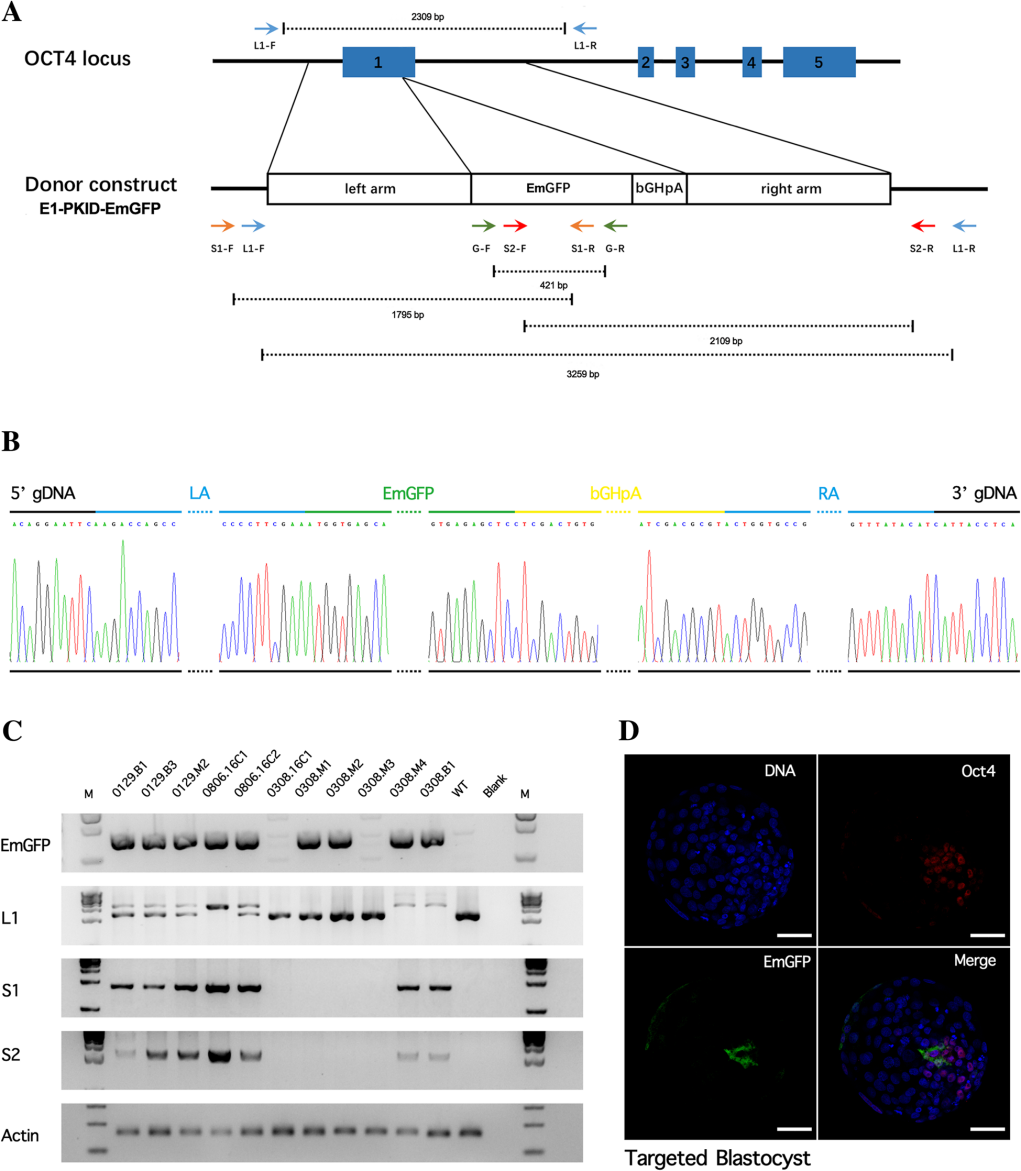
Genotyping and immunofluorescence of embryos generated by TALEN-mediated genome engineering. **a** Schematic overview of the strategy to generate an *OCT4-EmGFP* knock-in allele. The homologous arms of the donor vector are indicated as LA (839 bp) and RA (1001 bp). PCR primers used for PCR genotyping are shown as arrows in different colors. **b** PCR products obtained using primers L1-F and L1-R were sequenced. Sequence across the targeted region confirmed the correct fusion of EmGFP to the first exon of OCT4. **c** *EmGFP*: PCR genotyping using primers G-F and G-R produced bands of 421-bp fragment in the samples from nine embryos, suggesting the possible insertion or precise knock-in of the *EmGFP* gene. L1: PCR genotyping using primers L1-F and L1-R produced large products (3259 bp) in seven embryo samples, indicating the *EmGFP* sequence was integrated. The samples 0806.16C1, 0308 M4 and 0308.B1 only contain the larger product, suggesting either both alleles were targeted, or one allele failed to amplify. S1 and S2: PCR genotyping using primers S1-F and S1-R, S2-F and S2-R produced bands with correct size (1795 bp and 2109 bp) in the samples from the targeted embryos. **d** Immunostaining of targeted blastocysts using anti-GFP antibody showed a signal in the ICM. Scale bar, 50 μM

To determine whether the *EmGFP* sequence was inserted into the target *OCT4* locus, four primers (Fig. 4a, Table 2) were used to amplify the different PCR products from the individual embryos (*n* = 62) in the 16-cell, morula or blastocyst stages, and these genes were then sequenced. PCR using a pair of primers for *EmGFP* (G-F/R) amplified a specific band in the genomic DNA of the embryos (Fig. 4c), which suggested the possible insertion or precise knock-in of the *EmGFP* gene. Using the forward primer outside of the left homology arm (LA) region (L1-F) and the reverse primer outside the right homology arm (RA) region (L1-R), we detected a large PCR product (3259 kb) for the EmGFP-knock-in in the *OCT4* gene locus of seven embryos (Fig. 4c). The PCR products of samples 0806.16C1 and 0806.16C2 were sequenced, and both 5′ and 3′ boundaries between *OCT4* and *EmGFP* were detected as designed (Fig. 4b, Additional file 1). To further confirm the precise modification, we were then able to amplify a fragment of the expected size from the seven *EmGFP*-positive embryos using a forward primer outside of the LA region (S1-F) with an *EmGFP*-specific reverse primer (S1-R), and an *EmGFP*-specific forward primer (S2-F) with a reverse primer outside of the RA region (S2-R) (Fig. 4c). These results of electrophoresis and sequencing demonstrated that the *OCT4-EmGFP* knock-in allele has been successfully generated by TALENs-mediated HR at the efficiency of 11.3% (7 out of 62) in monkey embryos (Table 3).

**Table 3:** The positive rates of *EmGFP* knock-in demonstrated by PCR analysis or GFP detection

Method	Number of zygote injected with plasmid	Number of positive embryos	positive rate
PCR analysis	62	7	11.3%
GFP detection	9	1	11.1%

### Detection of OCT4 and GFP by immunofluorescence

In order to investigate the function of the knock-in *EmGFP* as a reporter for *OCT4* expression, *OCT4* and *GFP* were assayed by immunofluorescence in the individual blastocysts (*n* = 9). As expected based on a correctly targeted and functional allele, EmGFP expression was observed in the partial inner cell mass (ICM) region of the targeted blastocysts (Fig. 4d) at an efficiency of 11.1% (1 out of 9), corresponding with the value obtained by sequencing (Table 3). Besides, the proportion of the EmGFP-positive blastomere in OCT4-positive blastomere is 50% (11/20).These results indicated that, although with mosaicism, *EmGFP* was inserted into the desired site and could also successfully report the expression of *OCT4.*

### Assessment of the specificity of TALEN-mediated cleavage

To test the specificity of the cleavage by TALENs, seven highly-ranked potential off-target sites for *OCT4* TALENs were predicted and selected (Additional file 2: Table S1). The off-target effects were comprehensively assessed in the same way as the on-target effect, using genomic DNA from 10 embryos that were modified at the target site. Thus, the fragments around the seven potential off-target gene loci were amplified by PCR and sequenced. Sequencing results (Additional file 3) showed that no off-target mutations were found at the predicted sites of genomic DNA from the TALEN-injected embryos, suggesting that the TALENs we designed and constructed had high specificity and thus were sufficiently safe to use in monkeys.

## Discussion

With the emergence of reliable and precise gene editing tools in the recent years, gene modification has been dramatically brought to a new level. Among the engineered site-specific nucleases, CRISPR/Cas9 system has developed most rapidly and is now the most widely-used tool in research labs because of its construction simplicity and high efficiency. However, this system is still controversial in terms of safety because of the associated frequency of off-target effects [32, 33]. In comparison, TALEN is safer to use due to its requirement for being a dimer to be active and because it consists only of proteins. Therefore, TALEN is still an important and attractive genetic tool for functional genomics. In this study, we used the classic method of Golden Gate cloning for TAL assembly to construct a TALEN pair that could effectively work in both humans and monkeys at exon 1 of the *OCT4* gene (Fig. 2, Fig. 3).

Mammalian animal models have contributed significantly to studies in both basic biology and human diseases. In this regard, genome engineering in mammals is highly relevant, especially targeted gene knock-in methods. For example, allowing reporter genes to be successfully inserted into specific genes important in embryonic stem cell mechanisms, such as *OCT4, NANOG* and *SOX2*, can facilitate developmental biology research [27] [34]. Furthermore, insertion of certain functional genes can be helpful in the generation of animal models or in animal husbandry [3, 24]. In addition, gene knock-in could be used to replace the endogenous base sequences for repairing mutations. Many knock-in animals of different species have been generated to date. In this work, we obtained monkey embryos with precise *EmGFP* knock-in at the *OCT4* gene locus by TALEN-mediated HR at an efficiency of 11% (Fig. 4, Table 3), which corresponds to the general achievable efficiency of gene knock-in in mammalian systems [22] [35]. According to the results of immunofluorescence experiments, detection of GFP not only confirmed the expression of OCT4, but also reliably indicated the ICM regions of blastocysts. Therefore, this system could be a useful tool for embryonic development research in monkeys.

NHPs are the most suitable models to study human disease because of their high homology to humans. Our results are significant for the genetic modification research in monkeys, and are therefore highly valuable for the construction of reliable human disease models. Similar to the work of Yang and Niu groups on gene knock-in monkeys using CRISPR/Cas9 system [28, 29], our results confirm the successful gene insertion but show a degree of mosaicism, indicated by the immunofluorescence result (Fig. 4d) and electrophoretic images from four samples, 0129.B1, 0129.B3, 0129.M2 and 0806.16C2. Nevertheless, there are also three genetically modified samples (0806.16C1, 0308 M3 and 0308B1) that seem to be homozygous (Fig. 4c), encouraging us to undertake further study to overcome mosaicism in monkey genome editing.

Recently, J.R. et al. [36] combined 2C-HR-CRISPR with a modified biotin–streptavidin system to localize the repair templates to target sites, resulting in a more-than-tenfold increase (up to 95%) in knock-in efficiency over standard methods. Although their result was based on the CRISPR/Cas9 system unlike our study, it prompts us to carefully consider the time window for the injection of the TALEN pair and the donor template. The structure of chromatin is one of the determining factors for achieving high-efficiency of gene knock-in. In this research, we injected TALENs-coding plasmids and the donor vector 6–8 h after fertilization, when chromatin is most opened. Therefore, in the future, we need to find the optimum conditions of introducing the editing system to obtain more efficient gene knock-in results in monkeys.

## Conclusions

In this work, for the first time, we have successfully obtained *OCT4-EmGFP* knock-in monkey embryos by TALEN-assisted HR. Even though mosaicism was detected, the GFP could faithfully report the expression of OCT4. Our results suggest that gene targeting through HR mediated by TALENs is a feasible approach to introduce precise genetic modification in NHPs.

## Methods

### Construction of TALEN-coding plasmids and the donor plasmid

TALEs were designed using online software (https://tale-nt.cac.cornell.edu/node/add/talen). Repeat Variable Diresidue (RVD)-containing repeats were assembled using the Golden Gate TAL Assembly Kit, and then were sub-cloned into FokI nuclease expression vectors containing modified ELD/KKR domains, ptCMV-153/47-VR-HD (Addgene #50703). Assembled RVD repeats were as follows: HD HD HD HD NI NN NI NN HD HD HD NG NN HD NI HD for the left TALEN targeting CCCCAGAGCCCTGCAC; HD HD NG NG HD NG HD HD NI NN HD NG NG HD NI HD for the right TALEN targeting CCTTCTCCAGCTTCAC.

HR donor vector E1-PKID-EmGFP was constructed by standard molecular-cloning methods. For the *OCT4* locus, the donor vector consists of the 839-bp 5′-homology arm and the 1001-bp 3′-homology arm cloned from monkey (050139) gDNA, flanking an *EmGFP* cassette followed by the BGH polyA signal (Fig. 4, Additional file 4: Figure S1).

### SSA assay in human cells

For construction of the reporter plasmid pJL4-Maca-540, the 541-bp oligonucleotides near the TALENs target site was amplified with primer Maca-540-F1/R1 (Additional file 5) and cloned into the backbone vector pJL4-SSA(Cell Inspire Bio)linearized with BglII and EcoRI (NEB, R0144 and R0101). In this way the GFP-coding sequence could be separated into two parts, GF and FP. The two parts could be annealed to express intact GFP only following successful application of TALENs. The TALEN plasmids and the reporter plasmids were transfected into HEK293T cells using polyethylenimine (PEI,Polysciences 23,966–2) by applying standard protocol [37]. At 19 h and 47 h post transfection, respectively, the fluorescence intensity of GFP was measured using confocal microscopy.

### Animals

Healthy female cynomolgus monkeys (*Macaca fascicularis*), ranging in age from 5 to 8, were selected for use in this study. All animals were housed at Yunnan Key Laboratory of Primate Biomedical Research (LPBR). These animals were only used for oocytes collection. After a seven-day observation period, no euthanasia needed, these animals returned to the outdoor animal colony of LPBR.

### Embryo collection

Embryo collection and transfer were performed as previously described [38]. In brief, 11 healthy female cynomolgus monkeys aged 5–8 years with regular menstrual cycles were selected as oocyte donors for superovulation, which was performed by intramuscular injection with rhFSH (recombinant human follitropin alfa, GONAL-F, Merck Serono) for 8 days, then rhCG (recombinant human chorionic gonadotropin alfa, OVIDREL, Merck Serono) on day 9. 32–35 h after rhCG administration, Atropine(0.02 mg/kg,Tianjin Pharmaceutical Group, China), Benzyl penicillin sodium (20 mg/kg, Harbin Pharmaceutical Group, China) and morphine hydrochloride (0.2 mg/kg, Northeast Pharmaceutical Group, China) were intramuscular administered for preparing for minimally invasive surgery. Animals were anesthetized using Ketamine (10 mg/kg, Jiangsu Zhongmu Beikang Pharmaceutical CO., LTD, China).Oocytes were collected by laparoscopic follicular aspiration. Mature oocytes(MII oocytes,first polar body present) were used to perform intracytoplasmic sperm injection (ICSI) and the fertilization was confirmed by the presence of two pronuclei. The zygotes(including those subsequently microinjected with plasmids and) were cultured in the chemically defined, protein-free hamster embryo culture medium-10 (HECM-10) containing 10% fetal calf serum (Hyclone Laboratories, SH30088.02) at 37 °C in 5% CO2.

### Microinjection of plasmids

TALENs-coding plasmids (50 ng/μl) and donor plasmid (10 ng/μl) were mixed and microinjected into the cytoplasm of zygotes 6–8 h after fertilization using a microinjection system under standard conditions. The zygotes were then cultured in the chemically defined, protein-free hamster embryo culture medium-10 (HECM-10) containing 10% fetal calf serum (Hyclone Laboratories, SH30088.02) at 37 °C in 5% CO2.

### Genomic DNA extraction and genotyping by PCR

The individual embryos at the stage of 16-cell, morula or blastocyst were collected, and their genomic DNA was amplified using REPLI-g Single Cell Kit (QIAGEN, 150345). All PCR experiments were performed with Takara PrimeSTAR GXL DNA Polymerase (Takara, R050B), and the products were analyzed by sequencing. Primers OT-F/R were used to amplify the target site region at the following conditions: 95 °C for 5 min; 35 cycles of 95 °C for 30 s, 58 °C for 30 s, 72 °C for 1 min; 72 °C for 10 min. Primers G-F/R localizing to *EmGFP* were used to confirm possible insertion of *EmGFP*, under the following conditions: 95 °C for 5 min; 35 cycles of 95 °C for 30 s, 56 °C for 30 s, 72 °C for 30 s; 72 °C for 5 min. Primers S1-F/R amplified the fragment over outside the LA region and *EmGFP* under the conditions: 95 °C for 3 min; 16 cycles of 95 °C for 30 s, 66 °C for 30 s, 72 °C for 1.5 min, − 0.5 °C/cycle; 30 cycles of 95 °C for 30 s, 58 °C for 30s, 72 °C for 1.5 min; 72 °C for 5 min. Primers S2-F/R amplified the fragment over outside the RA region and *EmGFP* under the conditions: 95 °C for 5 min; 35 cycles of 95 °C for 30 s, 54 °C for 30 s, 72 °C for 2 min; 72 °C for 5 min. Primers L-F/R detected the large PCR product for the intact *EmGFP* knock-in at the *OCT4* locus, under the following conditions: 95 °C for 5 min; 35 cycles of 95 °C for 30 s, 60 °C for 30 s, 72 °C for 3 min; 72 °C for 5 min. The detailed information for all the primers is given in Table 2.

### Immunofluorescence

Immunofluorescence experiments were performed as described previously (Zhou et al., 2006). After fixation in 4% paraformaldehyde in PBS for 30 min and permeabilization in 0.2% Triton X-100/PBS for 45 min at room temperature, all embryo samples were blocked in PBS buffer containing 3% BSA for 45 min before incubation with the primary antibodies: anti-GFP antibody (1:1200, Abcam,ab13970) and anti-OCT4 antibody (1:100, Abcam,ab27985) overnight at 4 °C. After rinsing with PBS, embryos were incubated with the secondary antibodies and DAPI in PBS for 2 h at room temperature. EmGFP was labeled with Goat anti-Chicken IgY (H + L) Secondary Antibody, Alexa Fluor 488 (1:500, Invitrogen, A-11039). Endogenous OCT4 was stained by Donkey anti-Goat IgG (H + L) Cross-Adsorbed Secondary Antibody, Alexa Fluor 594 (1:500, Invitrogen, A-11058). DNA was counterstained with a DAPI solution (1:500, Solarbio, C0060). Finally, embryos were mounted on glass slides and examined using confocal microscopy.

### Off-target analysis

All potential off-target sites with a degree of homology to the pair of TALENs were predicted by Paired Target Finder in TAL Effector Nucleotide Targeter 2.0 [39] using NCBI GenBank assembly Mmul_051212 (GCA_000002255.2). We chose the 7 highest-ranked off-target sites for analysis. The fragments around these gene sites were amplified using the corresponding primers (Additional file 6) and analyzed by sequencing (Additional file 3).

## Additional files

Additional file 1: **Supplementary information 1.** Sequencing and Blast results of large PCR products of samples 0806.16C1 and 0806.16C2. The sequencing result indicated that *EmGFP* was successfully inserted into exon 1 of *OCT4* as expected. (PDF 1720 kb)

Additional file 2: **Table S1.** List of putative off-target sites. The top 7 potential off-target sites (OTS1-OTS7) were selected for analysis. (XLSX 15 kb)

Additional file 3: **Supplementary information 2**. Results of off-target sequencing analysis. (PDF 123 kb)

Additional file 4: **Figure S1.** Plasmid profile of the donor vector Donor-PKID-E1-EmGFP. (PDF 200 kb)

Additional file 5: **Supplementary information 3. **Construction of the reporter plasmid for the SSA assay. (PDF 226 kb)

Additional file 6: **Table S2.** Primers used for off-target analysis. (XLSX 13 kb)

## Abbreviations

ASD: Autism spectrum disorder
CRISPR/Cas9: Clustered regularly interspaced short palindromic repeats
DMD: Duchenne muscular dystrophy
DSBs: Double-strand breaks
HD: Huntington’s disease
HDR: Homology-directed repair
HR: Homologous recombination
ICM: Inner cell mass
NHP: Non-human primate
PD: Parkinson’s diseases
TALENs: Transcription activator-like effector nucleases
