# Supplementary material for: Homologous recombination-mediated targeted integration in monkey embryos using TALE nucleases
Source: BMC Biotechnol. 2019 Jan 15;19:7. doi: 10.1186/s12896-018-0494-2 (PMC6334428; doi:10.1186/s12896-018-0494-2)
Supplement: Supplementary file 1 — Supplementary information 1. Sequencing and Blast results of large PCR products of samples 0806.16C1 and 0806.16C2. The sequencing result indicated that EmGFP was successfully inserted into exon 1 of OCT4 as expected. (PDF 1720 kb) [file 12896_2018_494_MOESM1_ESM.pdf]

Sequence: OCT4-E1-EmGFP.dna (Circular / 3740 bp)
Enzymes: < Unsaved Enzyme Set > (16 of 646 total)
Features: 6 visible, 6 total

Unique Cutters **Bold**

10 20 30 40 50 60 70 80 90 100 110 120

5' CTTATGGCTGTTGATGCATTGAGGGATAGCTCCACACACACATTCAATAAATTTGAGGAGCTGAGAGGGTGACTGGCTCCCCGTGAAGGCACAGTGCCAGAGGTCTTTGGAGAGGGGGTG
3' GAATACCGACAACTACGTAACTCCCTATCGAGGTGTGTGTGTAAGTTATTTAAACTCCTCGACTCTCCCACTGACCGAGGGGCACTTCCGTGTCACGGTCTCCAGAAACCTCTCCCCCAC
5'-gDNA-out of LA

CTTATGGCTGTTGATGCATTGAGGGATAGCTCCACACACACATTCAATAAATTTGAGGAGCTGAGAGGGTGACTGGCTCCCCGTGAAGGCACAGTGCCAGAGGTCTTTGGAGAGGGGGTG

130 140 150 160 170 180 190 200 210 220 230 240

AAGCACCTGGGTTCCTGAAGAACATGGTGGTGTAGGAGTGATTCCAGACAGCTGGGATGTGCAGAGCCCGAGAGAGTGCCAGGGCGCAGGCTGGGAGTTGAAAGTTGGGTGTGGTGGCTC
TTCGTGGACCCAAGGACTTCTTGTACCACCACATCCTCACTAAGGTCTGTCGACCCTACACGTCTCGGGCTCTCTCACGGTCCCGCGTCCGACCCTCAACTTTCAACCCACACCACCGAG
5'-gDNA-out of LA

AAGCACCTGGGTTCCTGAAGAACATGGTGGTGTAGGAGTGATTCCAGACAGCTGGGATGTGCAGAGCCCGAGAGAGTGCCAGGGCGCAGGCTGGGAGTTGAAAGTTGGGTGTGGTGGCTC

250 260 270 280 290 300 310 320 330 340 350 360

ACGCCTTTAATCCTGACACTGGGAGGCAGAGGGGGGAGGATCGCTTGAGGACAGGAATTCAAGACCAGCCTGGGTAACATAGCAAGACCCCATCTCTACTAAAAATAAAAAAATCAACAG
TGCGGAAATTAGGACTGTGACCCTCCGTCTCCCCCCTCCTAGCGAACTCCTGTCCTTAAGTTCTGGTCGGACCCATTGTATCGTTCTGGGGTAGAGATGATTTTTATTTTTTTAGTTGTC
5'-gDNA-out of LA | E1-LA

ACGCCTTTAATCCTGACACTGGGAGGCAGAGGGGGGAGGATCGCTTGAGGACAGGAATTCAAGACCAGCCTGGGTAACATAGCAAGACCCCATCTCTACTAAAAATAAAAAAATCAACAG

1 ←
5' AGGATCGCTTGAGGACAGGAATTCAAGACCAGCCTGGGTAACATAGCAAGACCCCATCTCTACTAAAAATAAAAAAATCAACAG
3' TCCTAGCGAACTCCTGTCCTTAAGTTCTGGTCGGACCCATTGTATCGTTCTGGGGTAGAGATGATTTTTATTTTTTTAGTTGTC
5'-gDNA-out of LA | E1-LA

2 ←
5' GAGGATCGCTTGAGGACAGGAATTCAAGACCAGCCTGGGTAACATAGCAAGACCC-ATCTCTACTAAAAATAAAAAAATCAACAG
3' CTCCTAGCGAACTCCTGTCCTTAAGTTCTGGTCGGACCCATTGTATCGTTCTGGG-TAGAGATGATTTTTATTTTTTTAGTTGTC
5'-gDNA-out of LA | E1-LA

370 380 390 400 410 420 430 440 450 460 470 480

GTCGAGGTTGTCCAAGCCTGTAGTCCCAGCCACTCAGGAGGCTGGGGCAGAAGGATTGCTTCGGCCCAGTAGATCGAGGCTGCAATGAGCCAGCATTGTACCCCACTGCACTCCAGTCTG
CAGCTCCAACAGGTTCGGACATCAGGGTCGGTGAGTCCTCCGACCCCGTCTTCCTAACGAAGCCGGGTCATCTAGCTCCGACGTTACTCGGTCGTAACATGGGGTGACGTGAGGTCAGAC
E1-LA

GTCGAGGTTGTCCAAGCCTGTAGTCCCAGCCACTCAGGAGGCTGGGGCAGAAGGATTGCTTCGGCCCAGTAGATCGAGGCTGCAATGAGCCAGCATTGTACCCCACTGCACTCCAGTCTG

1 ←
GTCGAGGTTGTCCAAGCCTGTAGTCCCAGCCACTCAGGAGGCTGGGGCAGAAGGATTGCTTCGGCCCAGTAGATCGAGGCTGCAATGAGCCAGCATTGTACCCCACTGCACTCCAGTCTG
CAGCTCCAACAGGTTCGGACATCAGGGTCGGTGAGTCCTCCGACCCCGTCTTCCTAACGAAGCCGGGTCATCTAGCTCCGACGTTACTCGGTCGTAACATGGGGTGACGTGAGGTCAGAC
E1-LA

2 ←
GTCGAGGTTGTCCAAGCCTGTAGTCCCAGCCACTCAGGAGGCTGGGGCAGAAGGATTGCTTCGGCCCAGTAGATCGAGGCTGCAATGAGCCAGCATTGTACCC-ACTGCACTCCAGTCTG
CAGCTCCAACAGGTTCGGACATCAGGGTCGGTGAGTCCTCCGACCCCGTCTTCCTAACGAAGCCGGGTCATCTAGCTCCGACGTTACTCGGTCGTAACATGGG-TGACGTGAGGTCAGAC
E1-LA

490 500 510 520 530 540 550 560 570 580 590 600

PflFI
Tth111I

GGCAACAAAATAAGACCCTGTCTTAAAAAAAAAAA GGTTTCGGTGGGGACCTGTGCCGAGGTCCTGGAGGGGCGCCCAGTTGTGTCTCCGGTTGTCCCCTTCCACAGACACCACTGCC
CCGTTGTTTTATTCTGGGACAGAATTTTTTTTTTT CCAAAGCCACCCCTGGACACGGCTCCAGGACCTCCCCGCGGGTCAACACAGAGGCCAACAGGGGAAGGTGTCTGTGGTGACGG
E1-LA

GGCAACAAAATAAGACCCTGTCTTAAAAAAAAAAA GGTTTCGGTGGGGACCTGTGCCGAGGTCCTGGAGGGGCGCCCAGTTGTGTCTCCGGTTGTCCCCTTCCACAGACACCACTGCC

1 ←
PflFI
Tth111I
GGCAACAAAATAAGACCCTGTCTTAAAAAAAAAAAAGGTTTCGGTGGGGACCTGTGCCGAGGTCCTGGAGGGGCGCCCAGTTGTGTCTCCGGTTGTCCCCTTCCACAGACACCACTGCC
CCGTTGTTTTATTCTGGGACAGAATTTTTTTTTTTTCCAAAGCCACCCCTGGACACGGCTCCAGGACCTCCCCGCGGGTCAACACAGAGGCCAACAGGGGAAGGTGTCTGTGGTGACGG
E1-LA

2 ←
PflFI
Tth111I
GGCAACAAAATAAGACCCTGTCTTAAAAAAAAAAA GGTTTCGGTGGGGACCTGTGCCGAGGTCCTGGAGGGGCGCCCAGTTGTGTCTCCGGTTGTCCCCTTCCACAGACACCACTGCC
CCGTTGTTTTATTCTGGGACAGAATTTTTTTTTTT CCAAAGCCACCCCTGGACACGGCTCCAGGACCTCCCCGCGGGTCAACACAGAGGCCAACAGGGGAAGGTGTCTGTGGTGACGG
E1-LA

```
610 620 630 640 650 660 670 680 690 700 710 720

ACCACCATTAGGCAAACATCCTTCGCCTCAGTTTCTCCCCCCCACCTCCCTCTCCTCCACCCATCCAGGGGGCGGGGCCAGAGGTCAAGGCTAGTGGGTGGGACTGGGGAGGGAGAGAGG
TGGTGGTAATCCGTTTGTAGGAAGCGGAGTCAAAGAGGGGGGGTGGAGGGAGAGGAGGTGGGTAGGTCCCCCGCCCCGGTCTCCAGTTCCGATCACCCACCCTGACCCCTCCCTCTCTCC
E1-LA

ACCACCATTAGGCAAACATCCTTCGCCTCAGTTTCTCCCCCCCACCTCCCTCTCCTCCACCCATCCAGGGGGCGGGGCCAGAGGTCAAGGCTAGTGGGTGGGACTGGGGAGGGAGAGAGG
1 ←
ACCACCATTAGGCAAACATCCTTCGCCTCAGTTTCTCCCCCCCACCTCCCTCTCCTCCACCCATCCAGGGGGCGGGGCCAGAGGTCAAGGCTAGTGGGTGGGACTGGGGAGGGAGAGAGG
TGGTGGTAATCCGTTTGTAGGAAGCGGAGTCAAAGAGGGGGGGTGGAGGGAGAGGAGGTGGGTAGGTCCCCCGCCCCGGTCTCCAGTTCCGATCACCCACCCTGACCCCTCCCTCTCTCC
E1-LA
2 ←
ACCACCATTAGGCAAACATCCTTCGCCTCAGTTTCTCCCCCCCACCTCCCTCTCCTCCACCCATCCAGGGG-CGGGGCCAGAGGTCAAGGCTAGTGGGTGGGACTGGGGAGGGAGAGAGG
TGGTGGTAATCCGTTTGTAGGAAGCGGAGTCAAAGAGGGGGGGTGGAGGGAGAGGAGGTGGGTAGGTCCCC-GCCCCGGTCTCCAGTTCCGATCACCCACCCTGACCCCTCCCTCTCTCC
E1-LA
```

```
730 740 750 760 770 780 790 800 810 820 830 840

BtgI NcoI    PflMI*
GGTTGAGTAGTCCCTTAGCAAGCCCTCATTTCACCAGGCCCCCGGCTTGGGGTGCCTTCCTTCCCCATGGCGGGACACCTGGCTTCGGATTTCGCCTTCTCGCCCCCTCCAGGAGGTGGA
CCAACTCATCAGGGAATCGTTCGGGAGTAAAGTGGTCCGGGGGCCGAACCCCACGGAAGGAAGGGGTACCGCCCTGTGGACCGAAGCCTAAAGCGGAAGAGCGGGGGAGGTCCTCCACCT
E1-LA

GGTTGAGTAGTCCCTTAGCAAGCCCTCATTTCACCAGGCCCCCGGCTTGGGGTGCCTTCCTTCCCCATGGCGGGACACCTGGCTTCGGATTTCGCCTTCTCGCCCCCTCCAGGAGGTGGA
1 ←
BtgI NcoI    PflMI*
GGTTGAGTAGTCCCTTAGCAAGCCCTCATTTCACCAGGCCCCCGGCTTGGGGTGCCTTCCTTCCCCATGGCGGGACACCTGGCTTCGGATTTCGCCTTCTCGCCCCCTCCAGGAGGTGGA
CCAACTCATCAGGGAATCGTTCGGGAGTAAAGTGGTCCGGGGGCCGAACCCCACGGAAGGAAGGGGTACCGCCCTGTGGACCGAAGCCTAAAGCGGAAGAGCGGGGGAGGTCCTCCACCT
E1-LA
2 ←
BtgI NcoI    PflMI*
GGTTGAGTAGTCCCTTAGCAAGCCCTCATTTCACCAGGCCCCCGGCTTGGGGTGCCTTCCTTCCCCATGGCGGGACACCTGGCTTCGGATTTCGCCTTCTCGCCCCCTCCAGGAGGTGGA
CCAACTCATCAGGGAATCGTTCGGGAGTAAAGTGGTCCGGGGGCCGAACCCCACGGAAGGAAGGGGTACCGCCCTGTGGACCGAAGCCTAAAGCGGAAGAGCGGGGGAGGTCCTCCACCT
E1-LA
```

```
850 860 870 880 890 900 910 920 930 940 950 960

SfiI
GGTGATGGGCCAGGGGGGCCGGAGCCGGGCTGGGTTGATCCTCGGACCTGGCTAAGCTTCCAAGGCCCTCCTGGAGGGCCAGGAATCGGGCCGGGGGTTGGCCAGGCTCTGAGGTGTGG
CCACTACCCGGTCCCCCCGGCCTCGGCCCGACCCAACTAGGAGCCTGGACCGATTCGAAGGTTCCGGGAGGACCTCCCGGTCCTTAGCCCGGCCCCCAACCCGGTCCGAGACTCCACACC
E1-LA

GGTGATGGGCCAGGGGGGCCGGAGCCGGGCTGGGTTGATCCTCGGACCTGGCTAAGCTTCCAAGGCCCTCCTGGAGGGCCAGGAATCGGGCCGGGGGTTGGCCAGGCTCTGAGGTGTGG
1 ←
SfiI
GGTGATGGGCCAGGGGGGCCGGAGCCGGGCTGGGTTGATCCTCGGACCTGGCTAAGCTTCCAAGGCCCTCCTGGAGGGCCAGGAATCGGGCCGGGGGTTGGCCAGGCTCTGAGGTGTGG
CCACTACCCGGTCCCCCCGGCCTCGGCCCGACCCAACTAGGAGCCTGGACCGATTCGAAGGTTCCGGGAGGACCTCCCGGTCCTTAGCCCGGCCCCCAACCCGGTCCGAGACTCCACACC
E1-LA
2 ←
SfiI
GGTGATGGGCCAGGGGGGCCGGAGCCGGGCTGGGTTGATCCTCGGACCTGGCTAAGCTTCCAAGGCCCTCCTGGAGGGCCAGGAATCGGGCCGGGGGTTGGCCAGGCTCTGAGGTGTGG
CCACTACCCGGTCCCCCCGGCCTCGGCCCGACCCAACTAGGAGCCTGGACCGATTCGAAGGTTCCGGGAGGACCTCCCGGTCCTTAGCCCGGCCCCCAACCCGGTCCGAGACTCCACACC
E1-LA
```

```
970 980 990 1000 1010 1020 1030 1040 1050 1060 1070 1080

GGGATTCCCCCGTGCCCCCCGCCATATGAGTTCTGTGGGGGGATGGCGTACTGTGGGCCTCAGGTTGGAGTGGGGCTAGTGCCCCAAGGTGGCTTGGAGACCTCTCAGCCTGAGGGCGAA
CCCTAAGGGGGCACGGGGGGCGGTATACTCAAGACACCCCCCTACCGCATGACACCCGGAGTCCAACCTCACCCCGATCACGGGGTTCCACCGAACCTCTGGAGAGTCGGACTCCCGCTT
E1-LA

GGGATTCCCCCGTGCCCCCCGCCATATGAGTTCTGTGGGGGGATGGCGTACTGTGGGCCTCAGGTTGGAGTGGGGCTAGTGCCCCAAGGTGGCTTGGAGACCTCTCAGCCTGAGGGCGAA
1 ←
GGGATTCCCCCGTGCCCCCCGCCATATGAGTTCTGTGGGGGGATGGCGTACTGTGGGCCTCAGGTTGGAGTGGGGCTAGTGCCCCAAGGTGGCTTGGAGACCTCTCAGCCTGAGGGCGAA
CCCTAAGGGGGCACGGGGGGCGGTATACTCAAGACACCCCCCTACCGCATGACACCCGGAGTCCAACCTCACCCCGATCACGGGGTTCCACCGAACCTCTGGAGAGTCGGACTCCCGCTT
E1-LA
2 ←
GGGATTCCCC-GTGCCCCCCGCCATATGAGTTCTGTGGGGGGATGGCGTACTGTGGGCCTCAGGTTGGAGTGGGGCTAGTGCCCCAAGGTGGCTTGGAGACCTCTCAGCCTGAGGGCGAA
CCCTAAGGGG-CACGGGGGGCGGTATACTCAAGACACCCCCCTACCGCATGACACCCGGAGTCCAACCTCACCCCGATCACGGGGTTCCACCGAACCTCTGGAGAGTCGGACTCCCGCTT
E1-LA
```

```
610 620 630 640 650 660 670 680 690 700 710 720

ACCACCATTAGGCAAACATCCTTCGCCTCAGTTTCTCCCCCCCACCTCCCTCTCCTCCACCCATCCAGGGGGCGGGGCCAGAGGTCAAGGCTAGTGGGTGGGACTGGGGAGGGAGAGAGG
TGGTGGTAATCCGTTTGTAGGAAGCGGAGTCAAAGAGGGGGGGTGGAGGGAGAGGAGGTGGGTAGGTCCCCCGCCCCGGTCTCCAGTTCCGATCACCCACCCTGACCCCTCCCTCTCTCC
E1-LA

ACCACCATTAGGCAAACATCCTTCGCCTCAGTTTCTCCCCCCCACCTCCCTCTCCTCCACCCATCCAGGGGGCGGGGCCAGAGGTCAAGGCTAGTGGGTGGGACTGGGGAGGGAGAGAGG
1 ←
ACCACCATTAGGCAAACATCCTTCGCCTCAGTTTCTCCCCCCCACCTCCCTCTCCTCCACCCATCCAGGGGGCGGGGCCAGAGGTCAAGGCTAGTGGGTGGGACTGGGGAGGGAGAGAGG
TGGTGGTAATCCGTTTGTAGGAAGCGGAGTCAAAGAGGGGGGGTGGAGGGAGAGGAGGTGGGTAGGTCCCCCGCCCCGGTCTCCAGTTCCGATCACCCACCCTGACCCCTCCCTCTCTCC
E1-LA

2 ←
ACCACCATTAGGCAAACATCCTTCGCCTCAGTTTCTCCCCCCCACCTCCCTCTCCTCCACCCATCCAGGGG-CGGGGCCAGAGGTCAAGGCTAGTGGGTGGGACTGGGGAGGGAGAGAGG
TGGTGGTAATCCGTTTGTAGGAAGCGGAGTCAAAGAGGGGGGGTGGAGGGAGAGGAGGTGGGTAGGTCCCC-GCCCCGGTCTCCAGTTCCGATCACCCACCCTGACCCCTCCCTCTCTCC
E1-LA

730 740 750 760 770 780 790 800 810 820 830 840

BtgI
NcoI                                                                                     PflMI*
GGTTGAGTAGTCCCTTAGCAAGCCCTCATTTCACCAGGCCCCCGGCTTGGGGTGCCTTCCTTCCCCATGGCGGGACACCTGGCTTCGGATTTCGCCTTCTCGCCCCCTCCAGGAGGTGGA
CCAACTCATCAGGGAATCGTTCGGGAGTAAAGTGGTCCGGGGGCCGAACCCCACGGAAGGAAGGGGTACCGCCCTGTGGACCGAAGCCTAAAGCGGAAGAGCGGGGGAGGTCCTCCACCT
E1-LA

GGTTGAGTAGTCCCTTAGCAAGCCCTCATTTCACCAGGCCCCCGGCTTGGGGTGCCTTCCTTCCCCATGGCGGGACACCTGGCTTCGGATTTCGCCTTCTCGCCCCCTCCAGGAGGTGGA
1 ←
BtgI
NcoI                                                                                     PflMI*
GGTTGAGTAGTCCCTTAGCAAGCCCTCATTTCACCAGGCCCCCGGCTTGGGGTGCCTTCCTTCCCCATGGCGGGACACCTGGCTTCGGATTTCGCCTTCTCGCCCCCTCCAGGAGGTGGA
CCAACTCATCAGGGAATCGTTCGGGAGTAAAGTGGTCCGGGGGCCGAACCCCACGGAAGGAAGGGGTACCGCCCTGTGGACCGAAGCCTAAAGCGGAAGAGCGGGGGAGGTCCTCCACCT
E1-LA

2 ←
BtgI
NcoI                                                                                     PflMI*
GGTTGAGTAGTCCCTTAGCAAGCCCTCATTTCACCAGGCCCCCGGCTTGGGGTGCCTTCCTTCCCCATGGCGGGACACCTGGCTTCGGATTTCGCCTTCTCGCCCCCTCCAGGAGGTGGA
CCAACTCATCAGGGAATCGTTCGGGAGTAAAGTGGTCCGGGGGCCGAACCCCACGGAAGGAAGGGGTACCGCCCTGTGGACCGAAGCCTAAAGCGGAAGAGCGGGGGAGGTCCTCCACCT
E1-LA

850 860 870 880 890 900 910 920 930 940 950 960

SfiI
GGTGATGGGCCAGGGGGGCCGGAGCCGGGCTGGGTTGATCCTCGGACCTGGCTAAGCTTCCAAGGCCCTCCTGGAGGGCCAGGAATCGGGCCGGGGGTTGGGCCAGGCTCTGAGGTGTGG
CCACTACCCGGTCCCCCCGGCCTCGGCCCGACCCAACTAGGAGCCTGGACCGATTCGAAGGTTCCGGGAGGACCTCCCGGTCCTTAGCCCGGCCCCCAACCCGGTCCGAGACTCCACACC
E1-LA

GGTGATGGGCCAGGGGGGCCGGAGCCGGGCTGGGTTGATCCTCGGACCTGGCTAAGCTTCCAAGGCCCTCCTGGAGGGCCAGGAATCGGGCCGGGGGTTGGGCCAGGCTCTGAGGTGTGG
1 ←
SfiI
GGTGATGGGCCAGGGGGGCCGGAGCCGGGCTGGGTTGATCCTCGGACCTGGCTAAGCTTCCAAGGCCCTCCTGGAGGGCCAGGAATCGGGCCGGGGGTTGGGCCAGGCTCTGAGGTGTGG
CCACTACCCGGTCCCCCCGGCCTCGGCCCGACCCAACTAGGAGCCTGGACCGATTCGAAGGTTCCGGGAGGACCTCCCGGTCCTTAGCCCGGCCCCCAACCCGGTCCGAGACTCCACACC
E1-LA

2 ←
SfiI
GGTGATGGGCCAGGGGGGCCGGAGCCGGGCTGGGTTGATCCTCGGACCTGGCTAAGCTTCCAAGGCCCTCCTGGAGGGCCAGGAATCGGGCCGGGGGTTGGGCCAGGCTCTGAGGTGTGG
CCACTACCCGGTCCCCCCGGCCTCGGCCCGACCCAACTAGGAGCCTGGACCGATTCGAAGGTTCCGGGAGGACCTCCCGGTCCTTAGCCCGGCCCCCAACCCGGTCCGAGACTCCACACC
E1-LA

970 980 990 1000 1010 1020 1030 1040 1050 1060 1070 1080

GGGATTCCCCCGTGCCCCCCGCCATATGAGTTCTGTGGGGGGATGGCGTACTGTGGGCCTCAGGTTGGAGTGGGGCTAGTGCCCCAAGGTGGCTTGGAGACCTCTCAGCCTGAGGGCGAA
CCCTAAGGGGGCACGGGGGGCGGTATACTCAAGACACCCCCCTACCGCATGACACCCGGAGTCCAACCTCACCCCGATCACGGGGTTCCACCGAACCTCTGGAGAGTCGGACTCCCGCTT
E1-LA

GGGATTCCCCCGTGCCCCCCGCCATATGAGTTCTGTGGGGGGATGGCGTACTGTGGGCCTCAGGTTGGAGTGGGGCTAGTGCCCCAAGGTGGCTTGGAGACCTCTCAGCCTGAGGGCGAA
1 ←
GGGATTCCCCCGTGCCCCCCGCCATATGAGTTCTGTGGGGGGATGGCGTACTGTGGGCCTCAGGTTGGAGTGGGGCTAGTGCCCCAAGGTGGCTTGGAGACCTCTCAGCCTGAGGGCGAA
CCCTAAGGGGGCACGGGGGGCGGTATACTCAAGACACCCCCCTACCGCATGACACCCGGAGTCCAACCTCACCCCGATCACGGGGTTCCACCGAACCTCTGGAGAGTCGGACTCCCGCTT
E1-LA

2 ←
GGGATTCCCC-GTGCCCCCCGCCATATGAGTTCTGTGGGGGGATGGCGTACTGTGGGCCTCAGGTTGGAGTGGGGCTAGTGCCCCAAGGTGGCTTGGAGACCTCTCAGCCTGAGGGCGAA
CCCTAAGGGG-CACGGGGGGCGGTATACTCAAGACACCCCCCTACCGCATGACACCCGGAGTCCAACCTCACCCCGATCACGGGGTTCCACCGAACCTCTGGAGAGTCGGACTCCCGCTT
E1-LA
```

1090 1100 1110 1120 1130 1140 1150 1160 1170 1180 1190 1200

BstBI

GCAGGAGCCGGGGTGGAGAGCAACTCCGATGGGGCCTCCCCAGAGCCCTGCACCGTCCCCTTCGAAATGGTGAGCAAGGGCGAGGAGCTGTTCACCGGGGTGGTGCCCATCCTGGTCGAG
CGTCCTCGGCCCCACCTCTCGTTGAGGCTACCCCGGAGGGGTCTCGGGACGTGGCAGGGGAAGCTTTACCACTCGTTCCCGCTCCTCGACAAGTGGCCCCACCACGGGTAGGACCAGCTC

1 Met Val Ser Lys Gly 5 Glu Glu Leu Phe Thr 10 Gly Val Val Pro Ile 15 Leu Val Glu

E1-LA 1a EmGFP

GCAGGAGCCGGGGTGGAGAGCAACTCCGATGGGGCCTCCCCAGAGCCCTGCACCGTCCCCTTCGAAATGGTGAGCAAGGGCGAGGAGCTGTTCACCGGGGTGGTGCCCATCCTGGTCGAG

1

BstBI

GCAGGAGCCGGGGTGGAGAGCAACTCCGATGGGGCCTCCCCAGAGCCCTGCACCGTCCCCTTCGAAATGGTGAGCAAGGGCGAGGAGCTGTTCACCGGGGTGGTGCCCATCCTGGTCGAG
CGTCCTCGGCCCCACCTCTCGTTGAGGCTACCCCGGAGGGGTCTCGGGACGTGGCAGGGGAAGCTTTACCACTCGTTCCCGCTCCTCGACAAGTGGCCCCACCACGGGTAGGACCAGCTC

1 Met Val Ser Lys Gly 5 Glu Glu Leu Phe Thr 10 Gly Val Val Pro Ile 15 Leu Val Glu

E1-LA 1a EmGFP

2

BstBI

GCAGGAGCCGGGGTGGAGAGCAACTCCGATGGGGCCTCCCCAGAGCCCTGCACCGTCCCCTTCGAAATGGTGAGCAAGGGCGAGGAGCTGTTCACCGGGGTGGTGCCCATCCTGGTCGAG
CGTCCTCGGCCCCACCTCTCGTTGAGGCTACCCCGGAGGGGTCTCGGGACGTGGCAGGGGAAGCTTTACCACTCGTTCCCGCTCCTCGACAAGTGGCCCCACCACGGGTAGGACCAGCTC

1 Met Val Ser Lys Gly 5 Glu Glu Leu Phe Thr 10 Gly Val Val Pro Ile 15 Leu Val Glu

E1-LA 1a EmGFP

1210 1220 1230 1240 1250 1260 1270 1280 1290 1300 1310 1320

CTGGACGGCGACGTAAACGGCCACAAGTTCAGCGTGTCCGGCGAGGGCGAGGGCGATGCCACCTACGGCAAGCTGACCCTGAAGTTCATCTGCACCACCGGCAAGCTGCCCGTGCCCTGG
GACCTGCCGCTGCATTTGCCGGTGTTCAAGTCGCACAGGCCGCTCCCGCTCCCGCTACGGTGGATGCCGTTCGACTGGGACTTCAAGTAGACGTGGTGGCCGTTCGACGGGCACGGGACC

Leu Asp 20 Gly Asp Val Asn Gly 25 His Lys Phe Ser Val 30 Ser Gly Glu Gly Glu 35 Gly Asp Ala Thr Tyr 40 Gly Lys Leu Thr Leu 45 Lys Phe Ile Cys Thr 50 Thr Gly Lys Leu Pro 55 Val Pro Trp

EmGFP

CTGGACGGCGACGTAAACGGCCACAAGTTCAGCGTGTCCGGCGAGGGCGAGGGCGATGCCACCTACGGCAAGCTGACCCTGAAGTTCATCTGCACCACCGGCAAGCTGCCCGTGCCCTGG

1

CTGGACGGCGACGTAAACGGCCACAAGTTCAGCGTGTCCGGCGAGGGCGAGGGCGATGCCACCTACGGCAAGCTGACCCTGAAGTTCATCTGCACCACCGGCAAGCTGCCCGTGCCCTGG
GACCTGCCGCTGCATTTGCCGGTGTTCAAGTCGCACAGGCCGCTCCCGCTCCCGCTACGGTGGATGCCGTTCGACTGGGACTTCAAGTAGACGTGGTGGCCGTTCGACGGGCACGGGACC

Leu Asp 20 Gly Asp Val Asn Gly 25 His Lys Phe Ser Val 30 Ser Gly Glu Gly Glu 35 Gly Asp Ala Thr Tyr 40 Gly Lys Leu Thr Leu 45 Lys Phe Ile Cys Thr 50 Thr Gly Lys Leu Pro 55 Val Pro Trp

EmGFP

2

CTGGACGGCGACGTAAACGGCCACAAGTTCAGCGTGTCCGGCGAGGGCGAGGGCGATGCCACCTACGGCAAGCTGACCCTGAAGTTCATCTGCACCACCGGCAAGCTGCCCGTGCCCTGG
GACCTGCCGCTGCATTTGCCGGTGTTCAAGTCGCACAGGCCGCTCCCGCTCCCGCTACGGTGGATGCCGTTCGACTGGGACTTCAAGTAGACGTGGTGGCCGTTCGACGGGCACGGGACC

Leu Asp 20 Gly Asp Val Asn Gly 25 His Lys Phe Ser Val 30 Ser Gly Glu Gly Glu 35 Gly Asp Ala Thr Tyr 40 Gly Lys Leu Thr Leu 45 Lys Phe Ile Cys Thr 50 Thr Gly Lys Leu Pro 55 Val Pro Trp

EmGFP

1330 1340 1350 1360 1370 1380 1390 1400 1410 1420 1430 1440

CCCACCCTCGTGACCACCTTGACCTACGGCGTGCAGTGCTTCGCCCGCTACCCCGACCACATGAAGCAGCACGACTTCTTCAAGTCCGCCATGCCCGAAGGCTACGTCCAGGAGCGCACC
GGGTGGGAGCACTGGTGGAACTGGATGCCGCACGTCACGAAGCGGGCGATGGGGCTGGTGTACTTCGTCGTGCTGAAGAAGTTCAGGCGGTACGGGCTTCCGATGCAGGTCCTCGCGTGG

Pro 60 Thr Leu Val Thr Thr 65 Leu Thr Tyr Gly Val 70 Gln Cys Phe Ala Arg 75 Tyr Pro Asp His Met 80 Lys Gln His Asp Phe 85 Phe Lys Ser Ala Met 90 Pro Glu Gly Tyr Val 95 Gln Glu Arg Thr

EmGFP

CCCACCCTCGTGACCACCTTGACCTACGGCGTGCAGTGCTTCGCCCGCTACCCCGACCACATGAAGCAGCACGACTTCTTCAAGTCCGCCATGCCCGAAGGCTACGTCCAGGAGCGCACC

1

CCCACCCTCGTGACCACCTTGACCTACGGCGTGCAGTGCTTCGCCCGCTACCCCGACCACATGAAGCAGCACGACTTCTTCAAGTCCGCCATGCCCGAAGGCTACGTCCAGGAGCGCACC
GGGTGGGAGCACTGGTGGAACTGGATGCCGCACGTCACGAAGCGGGCGATGGGGCTGGTGTACTTCGTCGTGCTGAAGAAGTTCAGGCGGTACGGGCTTCCGATGCAGGTCCTCGCGTGG

Pro 60 Thr Leu Val Thr Thr 65 Leu Thr Tyr Gly Val 70 Gln Cys Phe Ala Arg 75 Tyr Pro Asp His Met 80 Lys Gln His Asp Phe 85 Phe Lys Ser Ala Met 90 Pro Glu Gly Tyr Val 95 Gln Glu Arg Thr

EmGFP

2

CCCACCCTCGTGACCACCTTGACCTACGGCGTGCAGTGCTTCGCCCGCTACCCCGACCACATGAAGCAGCACGACTTCTTCAAGTCCGCCATGCCCGAAGGCTACGTCCAGGAGCGCACC
GGGTGGGAGCACTGGTGGAACTGGATGCCGCACGTCACGAAGCGGGCGATGGGGCTGGTGTACTTCGTCGTGCTGAAGAAGTTCAGGCGGTACGGGCTTCCGATGCAGGTCCTCGCGTGG

Pro 60 Thr Leu Val Thr Thr 65 Leu Thr Tyr Gly Val 70 Gln Cys Phe Ala Arg 75 Tyr Pro Asp His Met 80 Lys Gln His Asp Phe 85 Phe Lys Ser Ala Met 90 Pro Glu Gly Tyr Val 95 Gln Glu Arg Thr

EmGFP

```
1450       1460       1470       1480       1490       1500       1510       1520       1530       1540       1550       1560
ATCTTCTTCAAGGACGACGGCAACTACAAGACCCGCGCCGAGGTGAAGTTCGAGGGCGACACCCTGGTGAACCGCATCGAGCTGAAGGGCATCGACTTCAAGGAGGACGGCAACATCCTG
TAGAAGAAGTTCCTGCTGCCGTTGATGTTCTGGGCGCGGCTCCACTTCAAGCTCCCGCTGTGGGACCACTTGGCGTAGCTCGACTTCCCGTAGCTGAAGTTCCTCCTGCCGTTGTAGGAC
    100            105            110            115            120            125            130            135
Ile Phe Phe Lys Asp Asp Gly Asn Tyr Lys Thr Arg Ala Glu Val Lys Phe Glu Gly Asp Thr Leu Val Asn Arg Ile Glu Leu Lys Gly Ile Asp Phe Lys Glu Asp Gly Asn Ile Leu
EmGFP
```

```
ATCTTCTTCAAGGACGACGGCAACTACAAGACCCGCGCCGAGGTGAAGTTCGAGGGCGACACCCTGGTGAACCGCATCGAGCTGAAGGGCATCGACTTCAAGGAGGACGGCAACATCCTG
1 ←
ATCTTCTTCAAGGACGACGGCAACTACAAGACCCGCGCCGAGGTGAAGTTCGAGGGCGACACCCTGGTGAACCGCATCGAGCTGAAGGGCATCGACTTCAAGGAGGACGGCAACATCCTG
TAGAAGAAGTTCCTGCTGCCGTTGATGTTCTGGGCGCGGCTCCACTTCAAGCTCCCGCTGTGGGACCACTTGGCGTAGCTCGACTTCCCGTAGCTGAAGTTCCTCCTGCCGTTGTAGGAC
    100            105            110            115            120            125            130            135
Ile Phe Phe Lys Asp Asp Gly Asn Tyr Lys Thr Arg Ala Glu Val Lys Phe Glu Gly Asp Thr Leu Val Asn Arg Ile Glu Leu Lys Gly Ile Asp Phe Lys Glu Asp Gly Asn Ile Leu
EmGFP
2 ←
ATCTTCTTCAAGGACGACGGCAACTACAAGACCCGCGCCGAGGTGAAGTTCGAGGGCGACACCCTGGTGAACCGCATCGAGCTGAAGGGCATCGACTTCAAGGAGGACGGCAACATCCTG
TAGAAGAAGTTCCTGCTGCCGTTGATGTTCTGGGCGCGGCTCCACTTCAAGCTCCCGCTGTGGGACCACTTGGCGTAGCTCGACTTCCCGTAGCTGAAGTTCCTCCTGCCGTTGTAGGAC
    100            105            110            115            120            125            130            135
Ile Phe Phe Lys Asp Asp Gly Asn Tyr Lys Thr Arg Ala Glu Val Lys Phe Glu Gly Asp Thr Leu Val Asn Arg Ile Glu Leu Lys Gly Ile Asp Phe Lys Glu Asp Gly Asn Ile Leu
EmGFP
```

```
1570       1580       1590       1600       1610       1620       1630       1640       1650       1660       1670       1680
GGGCACAAGCTGGAGTACAACTACAACAGCCACAAGGTCTATATCACCGCCGACAAGCAGAAGAACGGCATCAAGGTGAACTTCAAGACCCGCCACAACATCGAGGACGGCAGCGTGCAG
CCCGTGTTCGACCTCATGTTGATGTTGTCGGTGTTCCAGATATAGTGGCGGCTGTTCGTCTTCTTGCCGTAGTTCCACTTGAAGTTCTGGGCGGTGTTGTAGCTCCTGCCGTCGCACGTC
    140            145            150            155            160            165            170            175
Gly His Lys Leu Glu Tyr Asn Tyr Asn Ser His Lys Val Tyr Ile Thr Ala Asp Lys Gln Lys Asn Gly Ile Lys Val Asn Phe Lys Thr Arg His Asn Ile Glu Asp Gly Ser Val Gln
EmGFP
```

```
GGGCACAAGCTGGAGTACAACTACAACAGCCACAAGGTCTATATCACCGCCGACAAGCAGAAGAACGGCATCAAGGTGAACTTCAAGACCCGCCACAACATCGAGGACGGCAGCGTGCAG
1 ←
GGGCACAAGCTGGAGTACAACTACAACAGCCACAAGGTCTATATCACCGCCGACAAGCAGAAGAACGGCATCAAGGTGAACTTCAAGACCCGCCACAACATCGAGGACGGCAGCGTGCAG
CCCGTGTTCGACCTCATGTTGATGTTGTCGGTGTTCCAGATATAGTGGCGGCTGTTCGTCTTCTTGCCGTAGTTCCACTTGAAGTTCTGGGCGGTGTTGTAGCTCCTGCCGTCGCACGTC
    140            145            150            155            160            165            170            175
Gly His Lys Leu Glu Tyr Asn Tyr Asn Ser His Lys Val Tyr Ile Thr Ala Asp Lys Gln Lys Asn Gly Ile Lys Val Asn Phe Lys Thr Arg His Asn Ile Glu Asp Gly Ser Val Gln
EmGFP
2 ←
GGGCACAAGCTGGAGTACAACTACAACAGCCACAAGGTCTATATCACCGCCGACAAGCAGAAGAACGGCATCAAGGTGAACTTCAAGACCCGCCACAACATCGAGGACGGCAGCGTGCAG
CCCGTGTTCGACCTCATGTTGATGTTGTCGGTGTTCCAGATATAGTGGCGGCTGTTCGTCTTCTTGCCGTAGTTCCACTTGAAGTTCTGGGCGGTGTTGTAGCTCCTGCCGTCGCACGTC
    140            145            150            155            160            165            170            175
Gly His Lys Leu Glu Tyr Asn Tyr Asn Ser His Lys Val Tyr Ile Thr Ala Asp Lys Gln Lys Asn Gly Ile Lys Val Asn Phe Lys Thr Arg His Asn Ile Glu Asp Gly Ser Val Gln
EmGFP
```

```
1690       1700       1710       1720       1730       1740       1750       1760       1770       1780       1790       1800
CTCGCCGACCACTACCAGCAGAACACCCCCATCGGCGACGGCCCCGTGCTGCTGCCCGACAACCACTACCTGAGCACCCAGTCCGCCCTGAGCAAAGACCCCAACGAGAAGCGCGATCAC
GAGCGGCTGGTGATGGTCGTCTTGTGGGGGTAGCCGCTGCCGGGGCACGACGACGGGCTGTTGGTGATGGACTCGTGGGTCAGGCGGGACTCGTTTCTGGGGTTGCTCTTCGCGCTAGTG
    180            185            190            195            200            205            210            215
Leu Ala Asp His Tyr Gln Gln Asn Thr Pro Ile Gly Asp Gly Pro Val Leu Leu Pro Asp Asn His Tyr Leu Ser Thr Gln Ser Ala Leu Ser Lys Asp Pro Asn Glu Lys Arg Asp His
EmGFP
```

```
CTCGCCGACCACTACCAGCAGAACACCCCCATCGGCGACGGCCCCGTGCTGCTGCCCGACAACCACTACCTGAGCACCCAGTCCGCCCTGAGCAAAGACCCCAACGAGAAGCGCGATCAC
1 ←
CTCGCCGACCACTACCAGCAGAACACCCCCATCGGCGACGGCCCCGTGCTGCTGCCCGACAACCACTACCTGAGCACCCAGTCCGCCCTGAGCAAAGACCCCAACGAGAAGCGCGATCAC
GAGCGGCTGGTGATGGTCGTCTTGTGGGGGTAGCCGCTGCCGGGGCACGACGACGGGCTGTTGGTGATGGACTCGTGGGTCAGGCGGGACTCGTTTCTGGGGTTGCTCTTCGCGCTAGTG
    180            185            190            195            200            205            210            215
Leu Ala Asp His Tyr Gln Gln Asn Thr Pro Ile Gly Asp Gly Pro Val Leu Leu Pro Asp Asn His Tyr Leu Ser Thr Gln Ser Ala Leu Ser Lys Asp Pro Asn Glu Lys Arg Asp His
EmGFP
2 ←
CTCGCCGACCACTACCAGCAGAACACCCCCATCGGCGACGGCCCCGTGCTGCTGCCCGACAACCACTACCTGAGCACCCAGTCCGCCCTGAGCAAAGACCCCAACGAGAAGCGCGATCAC
GAGCGGCTGGTGATGGTCGTCTTGTGGGGGTAGCCGCTGCCGGGGCACGACGACGGGCTGTTGGTGATGGACTCGTGGGTCAGGCGGGACTCGTTTCTGGGGTTGCTCTTCGCGCTAGTG
    180            185            190            195            200            205            210            215
Leu Ala Asp His Tyr Gln Gln Asn Thr Pro Ile Gly Asp Gly Pro Val Leu Leu Pro Asp Asn His Tyr Leu Ser Thr Gln Ser Ala Leu Ser Lys Asp Pro Asn Glu Lys Arg Asp His
EmGFP
```

1810 1820 1830 1840 1850 1860 1870 1880 1890 1900 1910 1920

BsrGI

ATGGTCCTGCTGGAGTTCGTGACCGCCGCCGGGATCACTCTCGGCATGGACGAGCTGTACAAGTGAGAGCTCCTCGACTGTGCCTTCTAGTTGCCAGCCATCTGTTGTTTGCCCCTCCCC

TACCAGGACGACCTCAAGCACTGGCGGCGGCCCTAGTGAGAGCCGTACCTGCTCGACATGTTCACTCTCGAGGAGCTGACACGGAAGATCAACGGTCGGTAGACAACAAACGGGGAGGGG

220 225 230 235
Met Val Leu Leu Glu Phe Val Thr Ala Ala Gly Ile Thr Leu Gly Met Asp Glu Leu Tyr Lys

EmGFP

bGH poly(A) signal

ATGGTCCTGCTGGAGTTCGTGACCGCCGCCGGGATCACTCTCGGCATGGACGAGCTGTACAAGTGAGAGCTCCTCGACTGTGCCTTCTAGTTGCCAGCCATCTGTTGTTTGCCCCTCCCC

1 ←

BsrGI

ATGGTCCTGCTGGAGTTCGTGACCGCCGCCGGGATCACTCTCGGCATGGACGAGCTGTACAAGTGAGAGCTCCTCGACTGTGCCTTCTAGTTGCCAGCCATCTGTTGTTTGCCCCTCCCC

TACCAGGACGACCTCAAGCACTGGCGGCGGCCCTAGTGAGAGCCGTACCTGCTCGACATGTTCACTCTCGAGGAGCTGACACGGAAGATCAACGGTCGGTAGACAACAAACGGGGAGGGG

220 225 230 235
Met Val Leu Leu Glu Phe Val Thr Ala Ala Gly Ile Thr Leu Gly Met Asp Glu Leu Tyr Lys

EmGFP

bGH poly(A) signal

2 ←

BsrGI

ATGGTCCTGCTGGAGTTCGTGACCGCCGCCGGGATCACTCTCGGCATGGACGAGCTGTACAAGTGAGAGCTCCTCGACTGTGCCTTCTAGTTGCCAGCCATCTGTTGTTTGCCCCTCCCC

TACCAGGACGACCTCAAGCACTGGCGGCGGCCCTAGTGAGAGCCGTACCTGCTCGACATGTTCACTCTCGAGGAGCTGACACGGAAGATCAACGGTCGGTAGACAACAAACGGGGAGGGG

220 225 230 235
Met Val Leu Leu Glu Phe Val Thr Ala Ala Gly Ile Thr Leu Gly Met Asp Glu Leu Tyr Lys

EmGFP

bGH poly(A) signal

1930 1940 1950 1960 1970 1980 1990 2000 2010 2020 2030 2040

CGTGCCTTCCTTGACCCTGGAAGGTGCCACTCCCACTGTCCTTTCCTAATAAAATGAGGAAATTGCATCGCATTGTCTGAGTAGGTGTCATTCTATTCTGGGGGGTGGGGTGGGGCAGGA

GCACGGAAGGAACTGGGACCTTCCACGGTGAGGGTGACAGGAAAGGATTATTTTACTCCTTTAACGTAGCGTAACAGACTCATCCACAGTAAGATAAGACCCCCCACCCCACCCCGTCCT

bGH poly(A) signal

CGTGCCTTCCTTGACCCTGGAAGGTGCCACTCCCACTGTCCTTTCCTAATAAAATGAGGAAATTGCATCGCATTGTCTGAGTAGGTGTCATTCTATTCTGGGGGGTGGGGTGGGGCAGGA

1 ←

CGTGCCTTCCTTGACCCTGGAAGGTGCCACTCCCACTGTCCTTTCCTAATAAAATGAGGAAATTGCATCGCATTGTCTGAGTAGGTGTCATTCTATTCTGGGGGGTGGGGTGGGGCAGGA

GCACGGAAGGAACTGGGACCTTCCACGGTGAGGGTGACAGGAAAGGATTATTTTACTCCTTTAACGTAGCGTAACAGACTCATCCACAGTAAGATAAGACCCCCCACCCCACCCCGTCCT

bGH poly(A) signal

2 ←

CGTGCCTTCCTTGACCCTGGAAGGTGCCACTCCCACTGTCCTTTCCTAATAAAATGAGGAAATTGCATCGCATTGTCTGAGTAGGTGTCATTCTATTCTGGGGGGTGGGGTGGGGCAGGA

GCACGGAAGGAACTGGGACCTTCCACGGTGAGGGTGACAGGAAAGGATTATTTTACTCCTTTAACGTAGCGTAACAGACTCATCCACAGTAAGATAAGACCCCCCACCCCACCCCGTCCT

bGH poly(A) signal

2050 2060 2070 2080 2090 2100 2110 2120 2130 2140 2150 2160

MluI

CAGCAAGGGGGAGGATTGGGAAGACAATAGCAGGCATGCGATCGACGCGTACTGGTGCCGTGAAGCTGGAGAAGGAGAAGCTGGAGCAAAACCCGGAGGAGGCAAGTGAGCTTCAATGGG

GTCGTTCCCCCTCCTAACCCTTCTGTTATCGTCCGTACGCTAGCTGCGCATGACCACGGCACTTCGACCTCTTCCTCTTCGACCTCGTTTTGGGCCTCCTCCGTTCACTCGAAGTTACCC

E1-RA

CAGCAAGGGGGAGGATTGGGAAGACAATAGCAGGCATGCGATCGACGCGTACTGGTGCCGTGAAGCTGGAGAAGGAGAAGCTGGAGCAAAACCCGGAGGAGGCAAGTGAGCTTCAATGGG

1 ←

MluI

CAGCAAGGGGGAGGATTGGGAAGACAATAGCAGGCATGCGATCGACGCGTACTGGTGCCGTGAAGCTGGAGAAGGAGAAGCTGGAGCAAAACCCGGAGGAGGCAAGTGAGCTTCAATGGG

GTCGTTCCCCCTCCTAACCCTTCTGTTATCGTCCGTACGCTAGCTGCGCATGACCACGGCACTTCGACCTCTTCCTCTTCGACCTCGTTTTGGGCCTCCTCCGTTCACTCGAAGTTACCC

E1-RA

2 ←

MluI

CAGCAAGGGGGAGGATTGGGAAGACAATAGCAGGCATGCGATCGACGCGTACTGGTGCCGTGAAGCTGGAGAAGGAGAAGCTGGAGCAAAACCCGGAGGAGGCAAGTGAGCTTCAATGGG

GTCGTTCCCCCTCCTAACCCTTCTGTTATCGTCCGTACGCTAGCTGCGCATGACCACGGCACTTCGACCTCTTCCTCTTCGACCTCGTTTTGGGCCTCCTCCGTTCACTCGAAGTTACCC

E1-RA

2170 2180 2190 2200 2210 2220 2230 2240 2250 2260 2270 2280

BstEII AarI

GTTGGGGTGTGGGGAGGTGGTCGTGACAGGGCAGCCTGATGGGGAAGTGGTCACCTGCAGCTGCCCATACCTGGCACCCAGGAGAGGAGCAGGCAGGGGCAGCTGTCTTGGCCTGGGAGG
CAACCCCACACCCCTCCACCAGCACTGTCCCGTCGGACTACCCCTTCACCAGTGGACGTCGACGGGTATGGACCGTGGGTCCTCTCCTCGTCCGTCCCCGTCGACAGAACCGGACCCTCC
E1-RA

GTTGGGGTGTGGGGAGGTGGTCGTGACAGGGCAGCCTGATGGGGAAGTGGTCACCTGCAGCTGCCCATACCTGGCACCCAGGAGAGGAGCAGGCAGGGGCAGCTGTCTTGGCCTGGGAGG

1

BstEII AarI

GTTGGGGTGTGGGGAGGTGGTCATGACAGGGCAGCCTGATGGGGAAGTGGTCACCTGCAGCTGCCCATACCTGGCACCCAGGAGAGGAGCAGGCAGGGGCAGCTGTCTTGGCCTGGGAGG
CAACCCCACACCCCTCCACCAGTACTGTCCCGTCGGACTACCCCTTCACCAGTGGACGTCGACGGGTATGGACCGTGGGTCCTCTCCTCGTCCGTCCCCGTCGACAGAACCGGACCCTCC
E1-RA

2

BstEII AarI

GTTGGGGTGTGGGGAGGTGGTCATGACAGGGCAGCCTGATGGGGAAGTGGTCACCTGCAGCTGCCCAGACCTGGCACCCAGGAGAGGAGCAGGCAGGGGCAGCTGTCTTGGCCTGGGAGG
CAACCCCACACCCCTCCACCAGTACTGTCCCGTCGGACTACCCCTTCACCAGTGGACGTCGACGGGTCTGGACCGTGGGTCCTCTCCTCGTCCGTCCCCGTCGACAGAACCGGACCCTCC
E1-RA

2290 2300 2310 2320 2330 2340 2350 2360 2370 2380 2390 2400

GGTGTGTATCGACTGTAGGTGAGGGCAGAGAGGCCTGGGAGAGTCGGAAGTTGGCCCAGGCTGGCCTTTGCTCTGGCCCAGCCCTTGTCAGGTTCTCTCACATTTCATAGGCCTGCCCAG
CCACACATAGCTGACATCCACTCCCGTCTCTCCGGACCCTCTCAGCCTTCAACCGGGTCCGACCGGAAACGAGACCGGGTCGGGAACAGTCCAAGAGAGTGTAAAGTATCCGGACGGGTC
E1-RA

GGTGTGTATCGACTGTAGGTGAGGGCAGAGAGGCCTGGGAGAGTCGGAAGTTGGCCCAGGCTGGCCTTTGCTCTGGCCCAGCCCTTGTCAGGTTCTCTCACATTTCATAGGCCTGCCCAG

1

GGTGTGTATCGACTGTAGGTGAGGGCAGAGAGGCCTGGGAGAGTCGGAAGTTGGCCCAGGCTGGCCTTTGCTCTGGCCCAGCCCTTGTCAGGTTCTCTCACATTTCATAGGCCTGCCCAG
CCACACATAGCTGACATCCACTCCCGTCTCTCCGGACCCTCTCAGCCTTCAACCGGGTCCGACCGGAAACGAGACCGGGTCGGGAACAGTCCAAGAGAGTGTAAAGTATCCGGACGGGTC
E1-RA

2

GGTGTGTATCGACTGTAGGTGAGGGCAGAGAGGCCTGGGAGAGTCGGAAGTTGGCCCAGGCTGGCCTTTGCTCTGGCCCAGCCCTTGTCAGGTTCTCTCACATTTCCTAGGCCTGCCCAG
CCACACATAGCTGACATCCACTCCCGTCTCTCCGGACCCTCTCAGCCTTCAACCGGGTCCGACCGGAAACGAGACCGGGTCGGGAACAGTCCAAGAGAGTGTAAAGGATCCGGACGGGTC
E1-RA

2410 2420 2430 2440 2450 2460 2470 2480 2490 2500 2510 2520

BglII
BstYI

GGTCAGGTCATTCATTACCGGCCCAGCACCAGACTCAGCTTGGGGTTGGTTTGAGCTCCTTTTCCCACCCTTAGTCCTGCTTGACCCTTATCAGACCCAAGATCTTGGCCTTAGGGTTAA
CCAGTCCAGTAAGTAATGGCCGGGTCGTGGTCTGAGTCGAACCCCAACCAAACTCGAGGAAAAGGGTGGGAATCAGGACGAACTGGGAATAGTCTGGGTTCTAGAACCGGAATCCCAATT
E1-RA

GGTCAGGTCATTCATTACCGGCCCAGCACCAGACTCAGCTTGGGGTTGGTTTGAGCTCCTTTTCCCACCCTTAGTCCTGCTTGACCCTTATCAGACCCAAGATCTTGGCCTTAGGGTTAA

1

BglII
BstYI

GGTCAGGTCATTCATTACCGGCCCAGCACCAGACTCAGCTTGGGGTTGGTTTGAGCTCCTTTTCCCACCCTTAGTCCTGCTTGACCCTTATCAGACCCAAGATCTTGGCCTTAGGGTTAA
CCAGTCCAGTAAGTAATGGCCGGGTCGTGGTCTGAGTCGAACCCCAACCAAACTCGAGGAAAAGGGTGGGAATCAGGACGAACTGGGAATAGTCTGGGTTCTAGAACCGGAATCCCAATT
E1-RA

2

BglII
BstYI

GGTCAGGTCATTCATTACCGGCCCAGCACCAGACTCAGCTTGGG-TTGGTTTGAGCTCCTTTTCCCACCCTTAGTCCTGCTTGACCCTTATCAGACCCAAGATCTTGGCCTTAGGGTTAA
CCAGTCCAGTAAGTAATGGCCGGGTCGTGGTCTGAGTCGAACCC-AACCAAACTCGAGGAAAAGGGTGGGAATCAGGACGAACTGGGAATAGTCTGGGTTCTAGAACCGGAATCCCAATT
E1-RA

2530 2540 2550 2560 2570 2580 2590 2600 2610 2620 2630

BsaAI
PmlI

GCAGAGCCTGGGGATAAAAA CAGTGCTTATCCCAGGATTATTGTTTCTGAAAGTCTAGGGTGTGGCTCGTTTCTGATAGGGGCTCCTGTTTGGGCTGTGTGTGTGCACGTGTGGAGCTG
CGTCTCGGACCCCTATTTTT GTCACGAATAGGGTCCTAATAACAAAGACTTTCAGATCCCACACCGAGCAAAGACTATCCCCGAGGACAAACCCGACACACACACGTGCACACCTCGAC
E1-RA

GCAGAGCCTGGGGATAAAAA CAGTGCTTATCCCAGGATTATTGTTTCTGAAAGTCTAGGGTGTGGCTCGTTTCTGATAGGGGCTCCTGTTTGGGCTGTGTGTGTGCACGTGTGGAGCTG

1 ←

BsaAI
PmlI

GCAGAGCCTGGGGATAAAAA CAGTGCTTATCCCAGGATTATTGTTTCTGAAAGTCTAGGGTGTGGCTCGTTTCTGATAGGGGCTCCTGTTTGGGCTGTGTGTGTGCACGTGTGGAGCTG
CGTCTCGGACCCCTATTTTT GTCACGAATAGGGTCCTAATAACAAAGACTTTCAGATCCCACACCGAGCAAAGACTATCCCCGAGGACAAACCCGACACACACACGTGCACACCTCGAC
E1-RA

2 ←

BsaAI
PmlI

GCAGAGCCTGGGGATAAAAAACAGTGCTTATCCCAGGATTATTGTTTCTGAAAGTCTAGGGTGTGGCTCGTTTCTGATAGGGGCTCCTGTTTGGGCTGTGTGTGTGCACGTGTGGAGCTG
CGTCTCGGACCCCTATTTTTTGTCACGAATAGGGTCCTAATAACAAAGACTTTCAGATCCCACACCGAGCAAAGACTATCCCCGAGGACAAACCCGACACACACACGTGCACACCTCGAC
E1-RA

0 2650 2660 2670 2680 2690 2700 2710 2720 2730 2740 2750

GGTTTACCTGCAGTCAAGTATAGGGCTTGTCTTCCCTTGACCTGTGCCTCAGGCCAGTGACTTGCCACTGAGTTAAGGTGCACACCCTGGCACCCCTTGTAGAGAGCTGGATTTTGATTG
CCAAATGGACGTCAGTTCATATCCCGAACAGAAGGGAACTGGACACGGAGTCCGGTCACTGAACGGTGACTCAATTCCACGTGTGGGACCGTGGGGAACATCTCTCGACCTAAAACTAAC
E1-RA

GGTTTACCTGCAGTCAAGTATAGGGCTTGTCTTCCCTTGACCTGTGCCTCAGGCCAGTGACTTGCCACTGAGTTAAGGTGCACACCCTGGCACCCCTTGTAGAGAGCTGGATTTTGATTG

1 ←

GGTTTACCTGCAGTCAAGTATAGGGCTTGTCTTCCCTTGACCTGTGCCTCAGGCCAGTGACTTGCCACTGTGTTAAGGTGCACACCCTGGCACCCCTTGTAGAGAGCTGGATTTTGATTG
CCAAATGGACGTCAGTTCATATCCCGAACAGAAGGGAACTGGACACGGAGTCCGGTCACTGAACGGTGACACAATTCCACGTGTGGGACCGTGGGGAACATCTCTCGACCTAAAACTAAC
E1-RA

2 ←

GGTTTACCTGCAGTCAAGTATAGGGCTTGTCTTCCCTTGACCTGTGCCTCAGGCCAGTGACTTGCCACTGTGTTAAGGTGCACACCCTGGCACCCCTTGTAGAGAGCTGGATTTTGATTG
CCAAATGGACGTCAGTTCATATCCCGAACAGAAGGGAACTGGACACGGAGTCCGGTCACTGAACGGTGACACAATTCCACGTGTGGGACCGTGGGGAACATCTCTCGACCTAAAACTAAC
E1-RA

0 2770 2780 2790 2800 2810 2820 2830 2840 2850 2860 2870

ACTTCAGCCTCGGTTCCAAAGTTGTAAACAAGAAAAATGGTGAGAGATTTCTCCAGGCCATTCCCCAAATGTAGAGCTGCTGAGGGATTGAAGGCATCCAGCCCTGCTGAGGACTATTAA
TGAAGTCGGAGCCAAGGTTTCAACATTTGTTCTTTTTACCACTCTCTAAAGAGGTCCGGTAAGGGGTTTACATCTCGACGACTCCCTAACTTCCGTAGGTCGGGACGACTCCTGATAATT
E1-RA

ACTTCAGCCTCGGTTCCAAAGTTGTAAACAAGAAAAATGGTGAGAGATTTCTCCAGGCCATTCCCCAAATGTAGAGCTGCTGAGGGATTGAAGGCATCCAGCCCTGCTGAGGACTATTAA

1 ←

ACTTCAGCCTCGGTTCCAAAGTTGTAAACAAGAAAAATGGTGAGAGATTTCTCCAGGCCATTCCCCAAATGTAGAGCTGCTGAGGGATTGAAGGCATCCAGCCCTGCTGAGGACTATTAA
TGAAGTCGGAGCCAAGGTTTCAACATTTGTTCTTTTTACCACTCTCTAAAGAGGTCCGGTAAGGGGTTTACATCTCGACGACTCCCTAACTTCCGTAGGTCGGGACGACTCCTGATAATT
E1-RA

2 ←

ACTTCAGCCTCGGTTCCAAAGTTGTAAACAAGAAAAATGGTGAGAGATTTCTCCAGGCCATTCCCCAAATGTAGAGCTGCTGAGGGATTGAAGTCATCCAGCCCTGCTGAGGACTATTAA
TGAAGTCGGAGCCAAGGTTTCAACATTTGTTCTTTTTACCACTCTCTAAAGAGGTCCGGTAAGGGGTTTACATCTCGACGACTCCCTAACTTCAGTAGGTCGGGACGACTCCTGATAATT
E1-RA

0 2890 2900 2910 2920 2930 2940 2950 2960 2970 2980 2990

AGATGTATCTTCCAGTCCTTCAAGGCGACAAGTGTAAGCAATTAGAGATTAAGTACTAAGCCTTGAGACCTCACAAAAAGGTGTGACTGGTTTCTGGAGTGACCGAGAAGCCCCAGCCTC
TCTACATAGAAGGTCAGGAAGTTCCGCTGTTCACATTCGTTAATCTCTAATTCATGATTCGGAACTCTGGAGTGTTTTTCCACACTGACCAAAGACCTCACTGGCTCTTCGGGGTCGGAG
E1-RA

AGATGTATCTTCCAGTCCTTCAAGGCGACAAGTGTAAGCAATTAGAGATTAAGTACTAAGCCTTGAGACCTCACAAAAAGGTGTGACTGGTTTCTGGAGTGACCGAGAAGCCCCAGCCTC

1 ←

AGATGTATCTTCCAGTCCTTCAAGGCGACAAGTGTAAGCAATTAGAGATTAAGTACTAAGCCTTGAGACCTCACAAAAAGGTGTGACTGGTTTCTGGAGTGACCGAGAAGCCCCAGCCTC
TCTACATAGAAGGTCAGGAAGTTCCGCTGTTCACATTCGTTAATCTCTAATTCATGATTCGGAACTCTGGAGTGTTTTTCCACACTGACCAAAGACCTCACTGGCTCTTCGGGGTCGGAG
E1-RA

2 ←

AGATGTATCTTCCAGTCCTTCAAGGCGACAAGTGTAAGCAATTAGAGATTAAGTACTAAGCCTTGAGACCTCACAAAAAGGTGTGACTGGTTTCTGGAGTGACGGAGAAGCCCCAGCCTC
TCTACATAGAAGGTCAGGAAGTTCCGCTGTTCACATTCGTTAATCTCTAATTCATGATTCGGAACTCTGGAGTGTTTTTCCACACTGACCAAAGACCTCACTGCCTCTTCGGGGTCGGAG
E1-RA

0 3010 3020 3030 3040 3050 3060 3070 3080 3090 3100 3110

MfeI

```
TTTGCAGGAGGTCACTGCTGAGCCTTGAATGATAATGGCTGGCAATTGTGGTCCACTTCCTAAGTGCCTGGCTGTGTGCTCCGTTTATACATCATTACCTCATTAACCAGCACAAAATCT
AAACGTCCTCCAGTGACGACTCGGAACTTACTATTACCGACCGTTAACACCAGGTGAAGGATTCACGGACCGACACACGAGGCAAATATGTAGTAATGGAGTAATTGGTCGTGTTTTAGA
```

E1-RA 3'-gDNA-out of RA >

```
TTTGCAGGAGGTCACTGCTGAGCCTTGAATGATAATGGCTGGCAATTGTGGTCCACTTCCTAAGTGCCTGGCTGTGTGCTCCGTTTATACATCATTACCTCATTAACCAGCACAAAATCT
```

1 ←

MfeI

```
TTTGCAGGAGGTCACTGCTGAGCCTTGAATGATAATGGCTGGCAATTGTGGTCCACTTCCTAAGTGCCTGGCTGTGTGCTCCGTTTATACATCATTACCTCATTAACCAGCACAAAATCT
AAACGTCCTCCAGTGACGACTCGGAACTTACTATTACCGACCGTTAACACCAGGTGAAGGATTCACGGACCGACACACGAGGCAAATATGTAGTAATGGAGTAATTGGTCGTGTTTTAGA
```

E1-RA 3'-gDNA-out of RA >

2 ←

MfeI

```
TTTGCAGGAGGTCACTGCTGAGCCTTGAATGATAATGGCTGGCAATTGTGGTCCACTTCCTAAGTGCCTGGCTGTGTGCTCCGTTTATACATCATTACCTCATTAACCAGCACAAAATCT
AAACGTCCTCCAGTGACGACTCGGAACTTACTATTACCGACCGTTAACACCAGGTGAAGGATTCACGGACCGACACACGAGGCAAATATGTAGTAATGGAGTAATTGGTCGTGTTTTAGA
```

E1-RA 3'-gDNA-out of RA >

0 3130 3140 3150 3160 3170 3180 3190 3200 3210 3220 3230

```
CCTAGGGGGAGGTATTATTATCCTATTTAACGGGTTTTAACTGCTAAATGATGAAGCAAGGATTTGGACCGGTGTTTACTTTATTCCAAGTCCCCAAAATAGAATTTGGAAAATCTAAGA
GGATCCCCCTCCATAATAATAGGATAAATTGCCCAAAATTGACGATTTACTACTTCGTTCCTAAACCTGGCCACAAATGAAATAAGGTTCAGGGGTTTTATCTTAAACCTTTTAGATTCT
```

3'-gDNA-out of RA >

```
CCTAGGGGGAGGTATTATTATCCTATTTAACGGGTTTTAACTGCTAAATGATGAAGCAAGGATTTGGACCGGTGTTTACTTTATTCCAAGTCCCCAAAATAGAATTTGGAAAATCTAAGA
```

1 ←

```
CCTAGGGGGAGGTATTATTATCCTATTTAACGGGTTTTAACTGCTAAATGATGAAGCAAGGATTTGGACCGGTGTTTACTTTATTCCAAGTCCCCAAAATAGAATTTGGAAAATCTAAGA
GGATCCCCCTCCATAATAATAGGATAAATTGCCCAAAATTGACGATTTACTACTTCGTTCCTAAACCTGGCCACAAATGAAATAAGGTTCAGGGGTTTTATCTTAAACCTTTTAGATTCT
```

3'-gDNA-out of RA >

2 ←

```
CCTAGGGGGAGGTATTATTATCCTATTTAACGGGTTTTAACTGCTAAATGATGAAGCAAGGATTTGGACCGGTGTTTACTTTATTCCAAGTCCCCAAAATAGAATTTGGAAAATCTAAGA
GGATCCCCCTCCATAATAATAGGATAAATTGCCCAAAATTGACGATTTACTACTTCGTTCCTAAACCTGGCCACAAATGAAATAAGGTTCAGGGGTTTTATCTTAAACCTTTTAGATTCT
```

3'-gDNA-out of RA >

0 3250 3260 3270 3280 3290 3300 3310 3320 3330 3340 3350

```
TAGCAGAGGGCATTTACCAGTTTGAGTTATTGGCTGAGCAGAAGTTGGGAATGAAAACAGCCTATTTGAAATTGATATGATCAAGCACCCTTTGAGGCTTCAGGACTACAAAAAGGCCTT
ATCGTCTCCCGTAAATGGTCAAACTCAATAACCGACTCGTCTTCAACCCTTACTTTTGTCGGATAAACTTTAACTATACTAGTTCGTGGGAAACTCCGAAGTCCTGATGTTTTTCCGGAA
```

3'-gDNA-out of RA >

```
TAGCAGAGGGCATTTACCAGTTTGAGTTATTGGCTGAGCAGAAGTTGGGAATGAAAACAGCCTATTTGAAATTGATATGATCAAGCACCCTTTGAGGCTTCAGGACTACAAAAAGGCCTT
```

1 ←

```
TAGCAGAGGGCATTTACCAGTTTGAGTTATTGGCTGAGCAGAAGTTGGGAATGAAAACAGCCTATTTGAAATTGATATGATCAAGCACCCTTTGAGGCTTCAGGACTACAAAAAGGCCTT
ATCGTCTCCCGTAAATGGTCAAACTCAATAACCGACTCGTCTTCAACCCTTACTTTTGTCGGATAAACTTTAACTATACTAGTTCGTGGGAAACTCCGAAGTCCTGATGTTTTTCCGGAA
```

3'-gDNA-out of RA >

2 ←

```
TAGCAGAGGGCATTTACCAGTTTGAGTTATTGGCTGAGCAGAAGTTGGGAATGAAAACAGCCTATTTGAAATTGATATGATCAAGCACCCTTTGAGGCTTCAGGACTACAAAAAGGCCTT
ATCGTCTCCCGTAAATGGTCAAACTCAATAACCGACTCGTCTTCAACCCTTACTTTTGTCGGATAAACTTTAACTATACTAGTTCGTGGGAAACTCCGAAGTCCTGATGTTTTTCCGGAA
```

3'-gDNA-out of RA >

3360 3370 3380 3390 3400 3410 3420 3430 3440 3450 3460 3470

```
G ATTTTTCCTCACTAGCTGTGCTCCTCTGTCCCCTGGCAGCCTCACTGGCATGCCCCAGGGCTCAGTCCTTCAACCTCTGCTCAATCTACCCTTCCTTCCTCTCACCCACCCTCGAGG
C TAAAAAGGAGTGATCGACACGAGGAGACAGGGGACCGTCGGAGTGACCGTACGGGGTCCCGAGTCAGGAAGTTGGAGACGAGTTAGATGGGAAGGAAGGAGAGTGGGTGGGAGCTCC
```

3'-gDNA-out of RA >

```
G ATTTTTCCTCACTAGCTGTGCTCCTCTGTCCCCTGGCAGCCTCACTGGCATGCCCCAGGGCTCAGTCCTTCAACCTCTGCTCAATCTACCCTTCCTTCCTCTCACCCACCCTCGAGG
```

1 ←

```
GTT-TTTTTCCTCACTAGCTGTGCTCCTCTGTCCCCGGCAGCCTCACTGGCATGCCCCAGGGCTCAGTCCTTCAACCTCTGCTCAATCTACCCTTCCTTCCTCTCACCCACCCTCGAGG
CAA-AAAAAGGAGTGATCGACACGAGGAGACAGGGGGCCGTCGGAGTGACCGTACGGGGTCCCGAGTCAGGAAGTTGGAGACGAGTTAGATGGGAAGGAAGGAGAGTGGGTGGGAGCTCC
```

3'-gDNA-out of RA >

2 ←

```
G TTTTTTCCTCACTAGCTGTGCTCCTCTGTCCCCGGCAGCCTCACTGGCATGCCCCAGGGCTCAGTCCTTCAACCTCTGCTCAGTCTACCCTTCCTTCCTCTCACCCACCCTCGAGG
C AAAAAAGGAGTGATCGACACGAGGAGACAGGGGGCCGTCGGAGTGACCGTACGGGGTCCCGAGTCAGGAAGTTGGAGACGAGTCAGATGGGAAGGAAGGAGAGTGGGTGGGAGCTCC
```

3'-gDNA-out of RA >

```
3480      3490      3500      3510      3520      3530      3540      3550      3560      3570      3580      3590

CTTAAATGCCATTTAGACACTAGATGACTACCGCGTTTTCTGTCTCCTGTGATGGCTCCCTGAACTGCTCCACCCCGATCACCCAGTTGCTCAAGGCCAAGCCCAGTCATCCTCAGTTTC
GAATTTACGGTAAATCTGTGATCTACTGATGGCGCAAAAGACAGAGGACACTACCGAGGGACTTGACGAGGTGGGGCTAGTGGGTCAACGAGTTCCGGTTCGGGTCAGTAGGAGTCAAAG
3'-gDNA-out of RA

CTTAAATGCCATTTAGACACTAGATGACTACCGCGTTTTCTGTCTCCTGTGATGGCTCCCTGAACTGCTCCACCCCGATCACCCAGTTGCTCAAGGCCAAGCCCAGTCATCCTCAGTTTC

1 ←
CTTAAATGCCATTTAGACACTAGATGACTACCGCGTTTTCTGTCTCCTGTGATGGCTCC    3'
                                                               ...
GAATTTACGGTAAATCTGTGATCTACTGATGGCGCAAAAGACAGAGGACACTACCGAGG    5'
3'-gDNA-out of RA

2 ←
CTTAAATGCCATTTAGACACTAGATGACTACCGCGTTTTCTGTCTCCTGTGATGGC    3'
                                                            ...
GAATTTACGGTAAATCTGTGATCTACTGATGGCGCAAAAGACAGAGGACACTACCG    5'
3'-gDNA-out of RA

3600      3610      3620      3630      3640      3650      3660      3670      3680      3690      3700      3710

TTTCACTTCCTACATCCTATCCTTAGGAAATATCCTGAATCAATCACAACCTAACCCCCTACCCTCAGCCACCATCATCTCTGCTGGGATTACCGTAGTAGCTTCTCGAATTCTACTGCT
AAAGTGAAGGATGTAGGATAGGAATCCTTTATAGGACTTAGTTAGTGTTGGATTGGGGGATGGGAGTCGGTGGTAGTAGAGACGACCCTAATGGCATCATCGAAGAGCTTAAGATGACGA
3'-gDNA-out of RA

TTTCACTTCCTACATCCTATCCTTAGGAAATATCCTGAATCAATCACAACCTAACCCCCTACCCTCAGCCACCATCATCTCTGCTGGGATTACCGTAGTAGCTTCTCGAATTCTACTGCT

3720      3730      3740

TCCTCCCTACTGTCTGTGGCCAAC    3'
                            ...
AGGAGGGATGACAGACACCGGTTG    5'
3'-gDNA-out of RA

TCCTCCCTACTGTCTGTGGCCAAC
```

Original Sequence: OCT4-E1-EmGFP.dna

1: 16C1-L1 ←
3263 bases
2 .. 3262 (3226 mismatches, 2 gaps)

2: 16C2-L1 ←
3256 bases
4 .. 3256 (270 mismatches, 6 gaps)
