# Supplementary material for: Homologous recombination-mediated targeted integration in monkey embryos using TALE nucleases
Source: BMC Biotechnol. 2019 Jan 15;19:7. doi: 10.1186/s12896-018-0494-2 (PMC6334428; doi:10.1186/s12896-018-0494-2)
Supplement: Supplementary file 3 — Supplementary information 2. Results of off-target sequencing analysis. (PDF 123 kb) [file 12896_2018_494_MOESM3_ESM.pdf]

Additional file 3. Supplementary information 2

Result of off target Sequencing (OTS1-OTS7, red sequence means the reign of Folk I work on)

OTS1

```
OT 1.txt             CCCCAACACCCAACCCAGGCCTCCGGGGAGGTGAGGGGATGGAAGCTGGAGATGGAGCTCAATAAAAACTCTTGAAGAAT   240
0806.16C1_OT1-F_TSS20 --------------------------------------------------------------------------------   197
0806.16C2_OT1-F_TSS20 --------------------------------------------------------------------------------   194
0819.M1_OT1-F_TSS20  --------------------------------------------------------------------------------   196
0819.B1_OT1-F_TSS20  --------------------------------------------------------------------------------   194
0826.M1_OT1-F_TSS20  --------------------------------------------------------------------------------   195
0826.B1-OT1-F_TSS20  --------------------------------------------------------------------------------   194
0128.M1_OT1-F_TSS20  --------------------------------------------------------------------------------   197
0128.B1_OT1-F_TSS20  --------------t-----------------------------------------------------------------   196
0306.M1_OT1-F_TSS20  --------------------------------------------------------------------------------   190
0306.B1-OT1-F_TSS20  --------------t-----------------------------------------------------------------   190
```

OTS2

```
OT2.TXT              TCTCTGCCTCCACGGACCCCTGTACCCAGGAAACAGGAAACACAGGCAGGTGATGTGGGAGAAGGAGTGACTCAGAAGTT   400
0806.16C1_OT-3-F_TSS2 --------------------------------------------------------------------------------   358
0806.16C2_OT-3-F_TSS2 --------------------------------------------------------------------------------   358
0819.M1_OT-3-F_TSS2  --------------------------------------------------------------------------------   358
0819.B1_OT-3-F_TSS2  --------------------------------------------------------------------------------   357
0826.M1_OT-3-F_TSS2  --------------------------------------------------------------------------------   358
0826.B1_OT-3-F_TSS2  --------------------------------------------------------------------------------   358
0128.M1_OT-3-F_TSS2  --------------------------------------------------------------------------------   358
0128.B1_OT-3-F_TSS2  --------------------------------------------------------------------------------   358
0306.M1_OT-3-F_TSS2  --------------------------------------------------------------------------------   358
0306.B1_OT-3-F_TSS2  --------------------------------------------------------------------------------   358
```

OTS3

```
OT3.TXT              CCAGTGGTTTCTACATTCTCCCCAGAACCCTAGGGTTTTTGTGGATTCCATGTAAGGACTATTTGGGGAAGTTGGAGGAG   320
0806.16C1_OT-4-F_TSS2 ------------------------------------------a-------------------------------------   278
0806.16C2_OT-4-F_TSS2 ------------------------------------------a-------------------------------------   278
0819.M1_OT-4-F_TSS2  --------------------------------------------------------------------------------   273
0819.B1_OT-4-F_TSS2  --------------------------------------------------------------------------------   277
0826.M1_OT-4-F_TSS2  ------------------------------------------a------------------------a------------   276
0826.B1_OT-4-F_TSS2  --------------------------------------------------------------------------------   278
0128.M1_OT-4-F_TSS2  ------------------------------------------a-------------------------------------   281
0128.B1_OT-4-F_TSS2  ------------------------------------------a-------------------------------------   274
0306.M1_OT-4-F_TSS2  ------------------------------------------a-------------------------------------   277
0306.B1_OT-4-F_TSS2  ------------------------------------------a-------------------------------------   277
```

OTS4

```
OT4.txt              CTCTTTATCCAGCTTCTCAGTGATCCCCCAGAGACCCAGGCAATCCCAGTGGGGGGTTGGGGGAGCACGGCAAGTCAAC   440
0806.16C1_OT5-F_TSS20 --------------------------------------------------------------------------------   402
0806.16C2_OT5-F_TSS20 --------------------------------------------------------------------------------   402
```

```
 0819.M1_OT5-F_TSS20 --------------------------------------------------------------------------------   402
 0819.B1_OT5-F_TSS20 --------------------------------------------------------------------------------   402
 0826.M1_OT5-F_TSS20 --------------------------------------------------------------------------------   400
 0826.B1_OT5-F_TSS20 --------------------------------------------------------------------------------   401
 0128.M1_OT5-F_TSS20 --------------------------------------------------------------------------------   399
 0128.B1_OT5-F_TSS20 --------------------------------------------------------------------------------   402
 0306.M1_OT5-F_TSS20 --------------------------------------------------------------------------------   400
 0306.B1_OT5-F_TSS20 --------------------------------------------------------------------------------   402
```

OTS5

```
             OT5.TXT TGTTGCCAAGGGCTATATCCATTCCTACCCTACCACCTACACTCCTCCACAGCTCTCCTGTTCTGTGAAGCTGGGGATGG   360
0806.16C1_OT-6-F_TSS2 ---------------------------------t----------------------------------------------   322
0806.16C2_OT-6-F_TSS2 ---------------------------------t----------------------------------------------   321
  0819.M1_OT-6-F_TSS2 ---------------------------------t----------------------------------------------   320
  0819.B1_OT-6-F_TSS2 ---------------------------------t----------------------------------------------   307
  0826.M1_OT-6-F_TSS2 ---------------------------------t----------------------------------------------   318
  0826.B1_OT-6-F_TSS2 --------------------------------------------------------------------------------   321
  0128.M1_OT-6-F_TSS2 ---------------------------------t----------------------------------------------   319
  0128.B1_OT-6-F_TSS2 ---------------------------------t----------------------------------------------   321
  0306.M1_OT-6-F_TSS2 --------------------------------------------------------------------------------   309
  0306.B1_OT-6-F_TSS2 ---------------------------------t----------------------------------------------   321
```

OTS6

```
             OT6.TXT TCCTCTTCCTCCTCCAGCTTCCCTCTTGACTCACTGCCTAACTCTTACTCATCTTTCAGGTCTCTGGTTAAACGTCACCA   340
0806.16C1_OT-7-F_TSS2 --------------------------------------------------------------------------------   298
0806.16C2_OT-7-F_TSS2 --------------------------------------------------------------------------------   297
  0819.M1_OT-7-F_TSS2 --------------------------------------------------------------------------------   298
  0819.B1_OT-7-F_TSS2 --------------------------------------------------------------------------------   298
  0826.M1_OT-7-F_TSS2 --------------------------------------------------------------------------------   297
  0826.B1_OT-7-F_TSS2 --------------------------------------------------------------------------------   297
  0128.M1_OT-7-F_TSS2 --------------------------------------------------------------------------------   294
  0128.B1_OT-7-F_TSS2 --------------------------------------------------------------------------------   298
  0306.M1_OT-7-F_TSS2 --------------------------------------------------------------------------------   298
  0306.B1_OT-7-F_TSS2 --------------------------------------------------------------------------------   295
```

OTS7

```
             OT7.TXT GTCTAAATGTGTGTGTCCCTCCCCAAGTTCACTTGTGATAGTGGTATTAGGGGGTAGGGCCTTTGGGAGGTGATTTGGTC   260
0806.16C1_OT-8-F_TSS2 ---------------------------------------------------------------C----------------   219
0806.16C2_OT-8-F_TSS2 --------------------------------------------------------------------------------   218
  0819.M1_OT-8-F_TSS2 --------------------------------------------------------------------------------   217
  0819.B1_OT-8-F_TSS2 --------------------------------------------------------------------------------   219
  0826.M1_OT-8-F_TSS2 --------------------------------------------------------------------------------   218
  0826.B1_OT-8-F_TSS2 ---------------------------------------------------------------C----------------   215
  0128.M1_OT-8-F_TSS2 --------------------------------------------------------------------------------   219
  0128.B1_OT-8-F_TSS2 --------------------------------------------------------------------------------   218
  0306.M1_OT-8-F_TSS2 ---------------------------------------------------------------C----------------   223
  0306.B1_OT-8-F_TSS2 --------------------------------------------------------------------------------   219
```
