# Supplementary material for: Homologous recombination-mediated targeted integration in monkey embryos using TALE nucleases
Source: BMC Biotechnol. 2019 Jan 15;19:7. doi: 10.1186/s12896-018-0494-2 (PMC6334428; doi:10.1186/s12896-018-0494-2)
Supplement: Supplementary file 4 — Figure S1. Plasmid profile of the donor vector Donor-PKID-E1-EmGFP. (PDF 200 kb) [file 12896_2018_494_MOESM4_ESM.pdf]

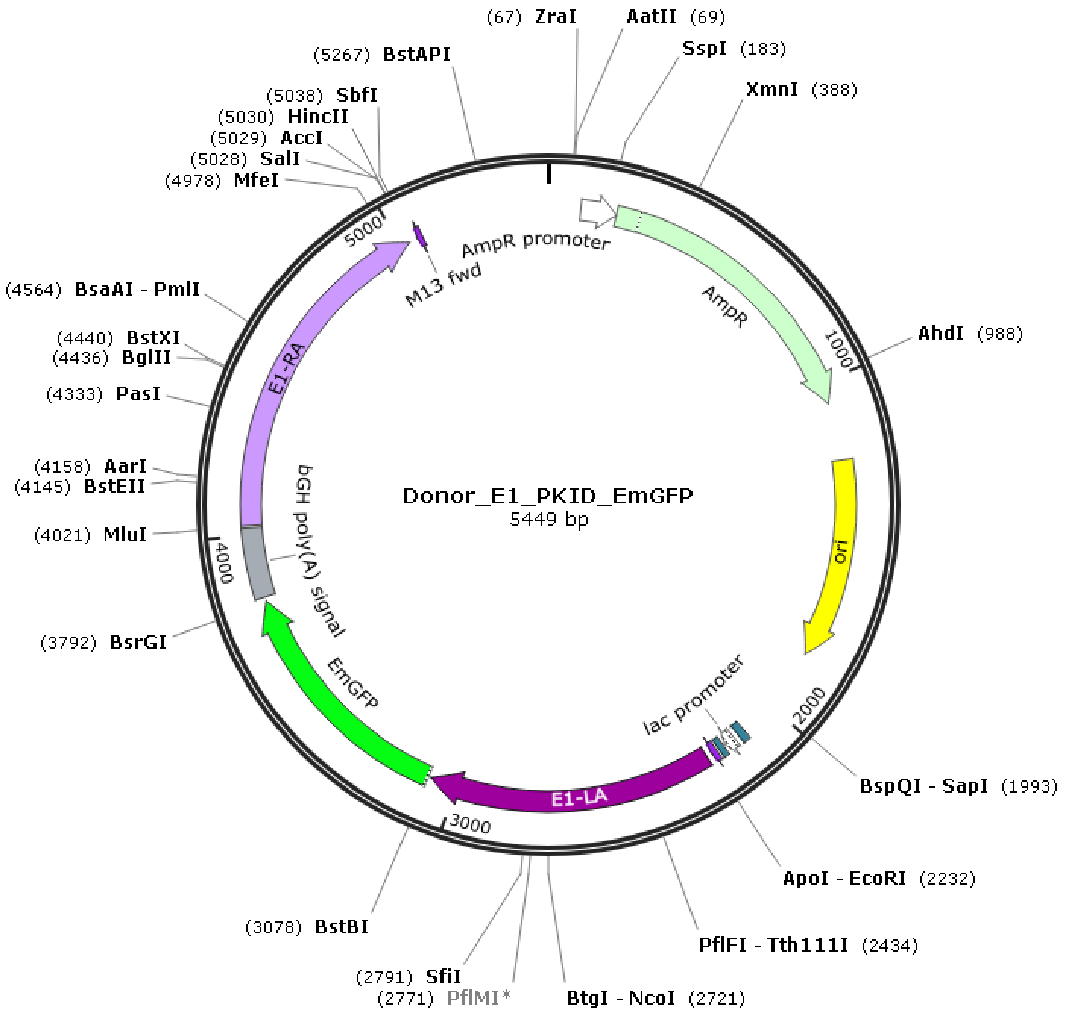

Figure S1. The plasmid profile of Donor vector Donor-PKID-E1-EmGFP. The insertion part, an EmGFP cassette followed by the BGHpolyA signal, is flanked by 5'-homology arm and the 3'-homology arm cloned from the monkey genome DNAs.
