# Supplementary material for: Homologous recombination-mediated targeted integration in monkey embryos using TALE nucleases
Source: BMC Biotechnol. 2019 Jan 15;19:7. doi: 10.1186/s12896-018-0494-2 (PMC6334428; doi:10.1186/s12896-018-0494-2)
Supplement: Supplementary file 5 — Supplementary information 3. Construction of the reporter plasmid for the SSA assay. (PDF 226 kb) [file 12896_2018_494_MOESM5_ESM.pdf]

Additional file 5: Supplementary information 3.

Oligonucleotides amplified from monkey's genome DNA for constructing the report plasmid pJL4-Maca-540 in SSA

TGATAAGGGTCAAGCAGGACTAAGGGTGGGAAAAGGAGCTCAAACCAACCCCAAGCTG
AGTCTGGTGCTGGGCCGGTAATGAATGACCTGACCCTGGGCAGGCCTATGAAATGTGAGA
GAACCTGACAAGGGCTGGGCCAGAGCAAAGGCCAGCCTGGGCCAACTTCCGACTCTCCCA
GGCCTCTCTGCCCTCACCTACAGTCGATACACACCCCTCCCAGGCCAAGACAGCTGCCCCT
GCCTGCTCCTCTCCTGGGTGCCAGGTATGGGCAGCTGCAGGTGACCACTTCCCCATCAGGC
TGCCCTGTCATGACCACCTCCCCACACCCCAACCCCATTGAAGCTCACTTGCCTCCTCCGG
GTTTTGCTCCAGCTTC**TCCTTCTCCAGCTTCAC**ggcaccagtggggacg**GTGCAGGGCTCTGGG**
GAGGCCCCATCGGAGTTGCTCTCCACCCCGGCTCCTGCTTCGCCCTCAGGCTGAGAGGTCT
CCAAGCCACCTTGGGGCACTAGCCCCACT**CCAACCTGAGGCCCACAGTACGCCAT**

Primers information for amplification

Maca-540-F1 CCG**GAATTCATGGCGTACTGTGGGCCTCAGGTTGG**
Maca-540-R1 **GAAGATCTTGATAAGGGTCAAGCAGGACTAAGGGTG**

Restriction Enzyme cutting site

AGATCT： Bgl II
GAATTC: EcoRI
